# Strikingly distinctive NH_3_-SCR behavior over Cu-SSZ-13 in the presence of NO_2_

**DOI:** 10.1038/s41467-022-32136-z

**Published:** 2022-08-08

**Authors:** Yulong Shan, Guangzhi He, Jinpeng Du, Yu Sun, Zhongqi Liu, Yu Fu, Fudong Liu, Xiaoyan Shi, Yunbo Yu, Hong He

**Affiliations:** 1 State Key Joint Laboratory of Environment Simulation and Pollution Control, Research Center for Eco-Environmental Sciences, Chinese Academy of Sciences, Beijing, 100085 China; 2 Center for Excellence in Regional Atmospheric Environment and Key Laboratory of Urban Pollutant Conversion, Institute of Urban Environment, Chinese Academy of Sciences, Xiamen, 361021 China; 3 University of Chinese Academy of Sciences, Beijing, 100049 China; 4 Department of Civil, Environmental, and Construction Engineering, Catalysis Cluster for Renewable Energy and Chemical Transformations (REACT), NanoScience Technology Center (NSTC), University of Central Florida, Orlando, FL 32816 USA

**Keywords:** Atmospheric chemistry, Pollution remediation, Heterogeneous catalysis

## Abstract

Commercial Cu-exchanged small-pore SSZ-13 (Cu-SSZ-13) zeolite catalysts are highly active for the standard selective catalytic reduction (SCR) of NO with NH_3_. However, their activity is unexpectedly inhibited in the presence of NO_2_ at low temperatures. This is strikingly distinct from the NO_2_-accelerated NO_*x*_ conversion over other typical SCR catalyst systems. Here, we combine kinetic experiments, in situ X-ray absorption spectroscopy, and density functional theory (DFT) calculations to obtain direct evidence that under reaction conditions, strong oxidation by NO_2_ forces Cu ions to exist mainly as Cu^II^ species (fw-Cu^2+^ and NH_3_-solvated Cu^II^ with high CNs), which impedes the mobility of Cu species. The SCR reaction occurring at these Cu^II^ sites with weak mobility shows a higher energy barrier than that of the standard SCR reaction on dynamic binuclear sites. Moreover, the NO_2_-involved SCR reaction tends to occur at the Brønsted acid sites (BASs) rather than the Cu^II^ sites. This work clearly explains the strikingly distinctive selective catalytic behavior in this zeolite system.

## Abstract (web summary)

Cu-SSZ-13 zeolites are highly active for standard NH_3_-SCR, but their activity is unexpectedly inhibited in the presence of NO_2_. This work demonstrates that strong oxidation by NO_2_ forces Cu ions to exist mainly as Cu^II^ species with low mobility, which is responsible for this distinctive behavior.

## Introduction

Increasingly stringent mobile source emission regulations have been pursued around the world to tackle environmental pollution. Nitrogen oxides (NO_*x*_) are inevitable gaseous pollutants emitted from internal combustion engines. Selective catalytic reduction of NO_*x*_ with NH_3_ (NH_3_-SCR) is the most widely adopted technology for the removal of NO_*x*_ from diesel engines^1,2^. The successful commercialization of Cu-SSZ-13 as an NH_3_-SCR catalyst is a significant achievement for diesel engine exhaust post treatment^3^. In the past decade, numerous studies have endeavored to uncover the standard SCR (SSCR) reaction mechanism^4–7^, hydrothermal deactivation mechanism^8–11^, and SO_2_ poisoning deactivation mechanism^12–14^, and to develop economic and sustainable synthesis methods for Cu-SSZ-13^15–18^, bringing about continuous optimization of Cu-SSZ-13 for commercial SCR catalysts.

In actual application, a diesel oxidation catalyst (DOC) is utilized to oxidize carbon monoxide (CO) and hydrocarbons (HCs), accompanied by partial oxidation of NO to NO_2_. The formed NO_2_ can participate in the NH_3_-SCR process through the so-called “fast SCR” reaction (FSCR, reaction 1, consisting of reactions 2 and 3). It is generally believed that the deNO_*x*_ efficiency of the FSCR reaction should be higher than that of SSCR (reaction 4) due to bypassing the oxidation of NO, which is usually the rate-limiting step in the SSCR reaction on V-based and Fe-zeolite catalysts^19,20^.

1 $$NO+N{O}_{2}+2N{H}_{3}\to 2{N}_{2}+3{H}_{2}O$$

2 $$2N{O}_{2}+2N{H}_{3}\to N{H}_{4}N{O}_{3}+{N}_{2}+{H}_{2}O$$

3 $$NO+N{H}_{4}N{O}_{3}\to {N}_{2}+N{O}_{2}+2{H}_{2}O$$

4 $$4NO+4N{H}_{3}+{O}_{2}\to 4{N}_{2}+6{H}_{2}O$$

However, there have been few studies reporting that NO_2_ measurably promotes the NH_3_-SCR efficiency over Cu-SSZ-13 catalytic systems. On the contrary, inhibition of NO conversion by NO_2_ was found over Al-rich Cu-SSZ-13 catalysts due to NH_4_NO_3_ formation, which is the so-called “abnormal fast NH_3_-SCR reaction”^21^. In our recent study, we found that the inhibiting effect of NO_2_ was closely related to Brønsted acid sites (BASs) and can be alleviated by hydrothermal aging due to the decrease in the number of BASs in Cu-SSZ-13^22^. Therefore, we speculated that NO_2_ reduction probably occurs at BASs. Also, we previously observed the reaction between NO and NH_4_NO_3_ occurring at BASs over the H-SSZ-13 catalyst^23^. Furthermore, Kubota et al. found that NO reacts with NH_4_NO_3_ more rapidly than NH_4_NO_3_ decomposition over H-AFX and H-CHA zeolites^24,25^. However, the situation in Cu-containing zeolites is more complicated. McEwen et al. found that four-fold-coordinated Cu(II) species dominate the Cu-SSZ-13 catalyst under FSCR conditions, which differs from the composition under SSCR conditions, where Cu(I) and Cu(II) species both exist^26^. Paolucci et al. investigated the oxidation process of Cu(I)(NH_3_)_2_ species by O_2_ and NO_2_. It was found that oxidation by NO_2_ occurred at isolated Cu sites, rather than at the Cu dimer sites required for O_2_ activation^5^. More recently, Liu et al. investigated the FSCR mechanism over the Cu-OH site on Cu-CHA zeolite and showed the important role of BASs in the FSCR reaction^27^. Therefore, it can be concluded that the FSCR reaction pathway over Cu-SSZ-13 is unique and different from other catalytic systems where NO_2_ accelerates SCR rates. The active sites as well as redox pathways may change over Cu-SSZ-13 in the presence of NO_2_. Compared to the relatively few studies on the FSCR reaction mechanism, researchers have conducted numerous experimental and theoretical studies to explore the SSCR reaction mechanism in the past decade. Thus, the SSCR mechanism has been relatively clear, in which dynamic binuclear Cu^+^ species are the primary active sites^4,5,28^. However, the influence of NO_2_ on the active Cu sites and the mechanism of the NO_2_-involved SCR reaction are barely discussed, and are worth exploring since NO and NO_2_ always coexist in actual applications.

In this study, the SCR reaction over the Cu-SSZ-13 catalyst in the presence of both NO and NO_2_ was studied by kinetic measurements. In situ X-ray absorption fine structure (XAFS) measurements were applied to reveal the state of copper species under SSCR (with only NO as NO_*x*_), FSCR (equal mixture of NO and NO_2_ as NO_*x*_) and NO_2_-SCR (only NO_2_ as NO_*x*_) reaction conditions. Density functional theory (DFT) calculations were conducted to identify the NO_2_-involved SCR reaction pathways. These results provide new insights into the role of NO_2_ in the NH_3_-SCR reaction and shed light on the actual application of Cu-SSZ-13 catalysts in the presence of both NO and NO_2_.

## Results and discussion

### Kinetic studies of NO_*x*_ conversion under SSCR, FSCR and NO_2_-SCR conditions

**Fig. 1:**
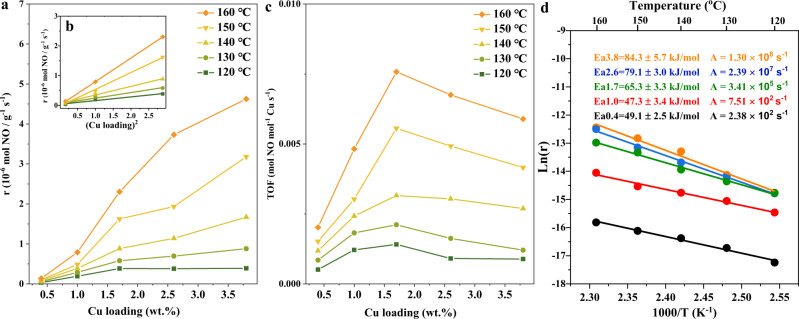
Kinetic analysis of standard SCR reaction. **a** SSCR reaction rates as a function of Cu loading. **b** SSCR reaction rates as a function of the square of Cu loading. **c** SSCR turnover frequencies (TOF) as a function of Cu loading. **d** Activation energies (Ea) and pre-exponential factors (A) with different Cu loadings.

We first carried out kinetic studies on the SSCR reaction, with the results shown in Fig. 1 and Supplementary Fig. 1. The SSCR rate increases linearly with the square of Cu loading when the Cu loading is below 1.7 wt.% (magnified in Fig. 1b), indicating the participation of Cu pairs in the standard NH_3_-SCR reaction. Previous studies have reported that Cu^I^ dimers are formed with O_2_ activation in the oxidation half-cycle (Cu^I^→Cu^II^)^4,5^. Recently, Hu et al. also proposed a Cu^II^-pair-mediated low-temperature reduction half-cycle (Cu^II^→Cu^I^)^6^. Chen et al. also indicated the participation of Cu pairs in the reduction half-cycle^29^. Therefore, the formation of a Cu pair in the same cage is significantly important for the overall standard NH_3_-SCR reaction process. The increase trend slows down with further rise in the Cu loading. The turnover frequency (TOF) shows a volcano-type tendency, with a maximum at Cu loading of 1.7 wt.% (Fig. 1c). The increase in TOF at low Cu loading is attributed to the quadratic increase in the SSCR rate. At high Cu loading, however, the decline of TOF is probably due to the underutilization of the active Cu sites. According to the calculation method reported by Jones et al.^30^, every 2.4 and 3.5 CHA cages contain one Cu ion for Cu_3.8_-SSZ-13 and Cu_2.6_-SSZ-13 samples, respectively. The formed Cu-NH_3_ complex or dimer Cu species under SSCR conditions probably impede the access of reactants to the Cu ions deep inside the pores, causing inefficiency in the use of Cu ions^31^. The activation energy (Ea) and pre-exponential factor (A) both increase with the increase in Cu loading, which was also observed by Gao et al^31^. Recently, Krisha et al. reported that the Ea of Cu^I^ oxidation increased monotonically with Cu density in a fixed kinetic regime due to the non-mean-field behavior of Cu-SSZ-13 in the NH_3_-SCR reaction and that the Ea of Cu^II^ reduction was unchanged when the Cu load was higher than 0.69 wt.%^32^. On the other hand, the kinetic relevance of Cu^II^ reduction increased with increasing Cu ion density, the Ea of which was higher than that of Cu^I^ oxidation^30,32^. Therefore, the increase of the Ea in Cu^I^ oxidation and kinetic relevance of Cu^II^ reduction both contributed to the increase in the Ea of the SSCR reaction.

**Fig. 2:**
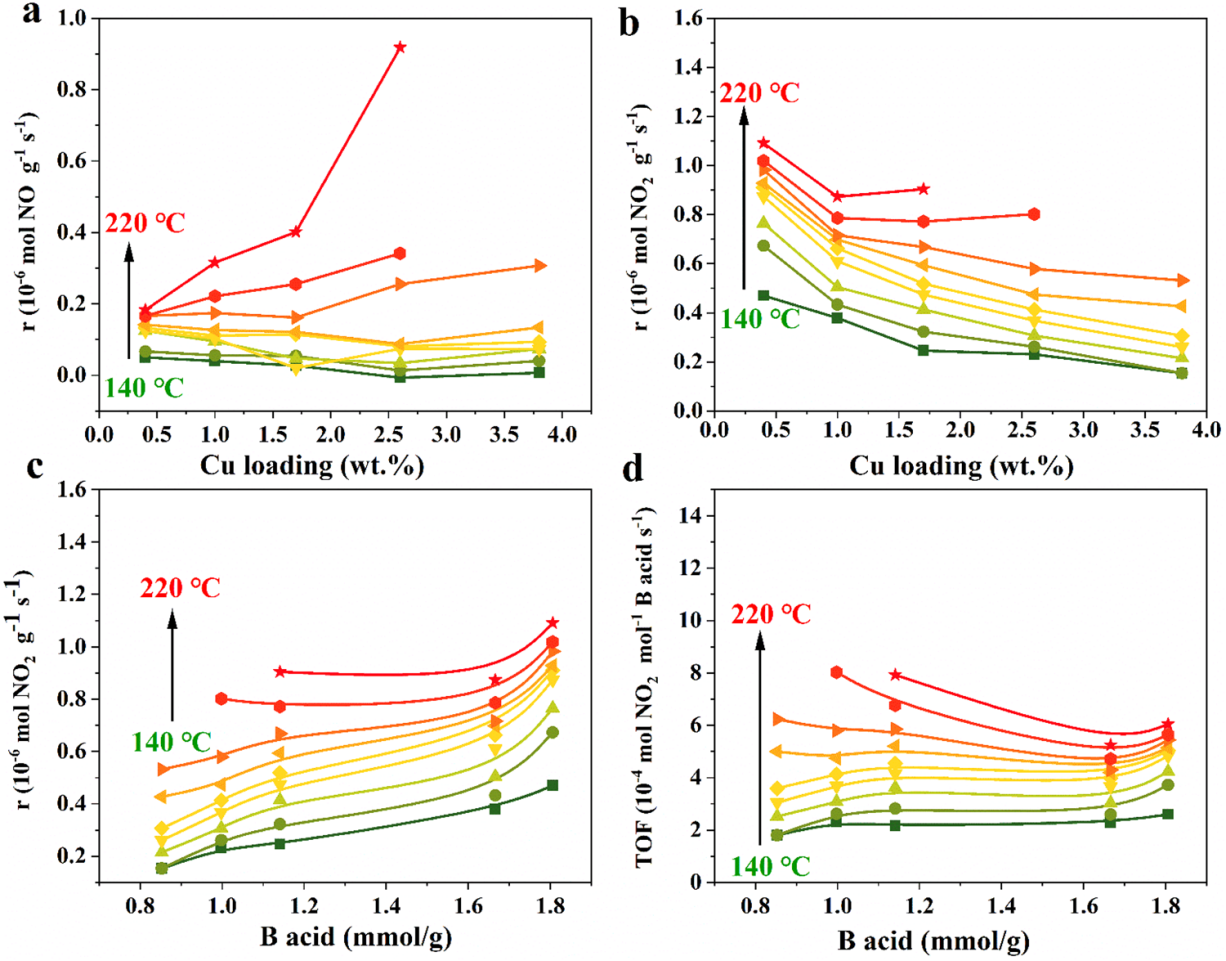
Kinetic analysis of fast SCR reaction. **a** NO reaction rates as a function of Cu loading over Cu-SSZ-13 catalysts in NO and NO_2_ gas mixtures. **b** NO_2_ reaction rates as a function of Cu loading over Cu-SSZ-13 in NO and NO_2_ gas mixtures. **c** NO_2_ reaction rates as a function of BASs over Cu-SSZ-13 in NO and NO_2_ gas mixtures. **d** NO_2_ turnover frequencies (TOFs) as a function of BASs in NO and NO_2_ gas mixtures.

Then, the FSCR reaction over Cu-SSZ-13 was carried out as shown in Supplementary Figs. 2a and 3a. Compared to the SSCR reaction, the NO_*x*_ conversion over Cu3.8-SSZ-13 was significantly inhibited in the presence of NO_2_, which was strikingly distinct from the NO_2_-accelerated NO_*x*_ conversion over Fe-based zeolite and oxide catalysts (Supplementary Fig. 3). Supplementary Fig. 2 shows the NO_*x*_, NO and NO_2_ conversion levels over Cu-SSZ-13 with different Cu loadings under steady-state FSCR conditions. We normalized the NO and NO_2_ reaction rates by the catalyst weight as a function of Cu loading, with the results shown in Fig. 2a, b, respectively. The NO consumption rates under FSCR and SSCR condition were compared (Supplementary Fig. 4) and the result showed that NO reduction was severely suppressed at low temperatures under FSCR conditions. The extremely low NO conversion at low temperatures was previously thought to be resulted from zeolite pore blocking by the formation of stable NH_4_NO_3_^21,23^. The NH_4_NO_3_ formation was verified by the observation of N_2_O in an FSCR-TPD experiment (Supplementary Fig. 5), since the N_2_O mainly originated from NH_4_NO_3_ decomposition. Interestingly, the NO_2_ reduction markedly decreased with the increase in Cu loading, while it increased as the number of BASs rose at low temperatures (Fig. 2b, c and Supplementary Fig. 6). This demonstrated that the block of active sites by NH_4_NO_3_ was not the only reason for the NO_2_-inhibition effects, otherwise both NO and NO_2_ reduction were inhibited. The BASs primarily participated in the reduction of NO_2_, which was also observed in the NO_2_-SCR reaction (Supplementary Fig. 7). Moreover, the turnover frequency (TOF) of NO_2_ on BASs hardly changed as the number of BAS varied. Supplementary Fig. 8 presents the NO_2_ reaction rate as a function of Cu loading and BASs under NO_2_-SCR conditions, which showed the same trend as that in the co-existence of NO and NO_2_. Moreover, we carried out the NO_2_-SCR reaction over H-SSZ-13 and Cu_2.6_-SSZ-13 with different Si/Al ratios and found that the zeolites with low Si/Al exhibited high NO_*x*_ conversion due to their high numbers of BASs at low temperatures (Supplementary Fig. 9). The above results indicated that NO_2_ primarily reacted at BASs while NO was difficult to be reduced in the presence of NO_2_. NO_2_ disproportionation occurs on the BASs to form nitrates and adsorbed NO^+^, which then react with NH_3_ to form NH_4_NO_3_ and NH_2_NO, respectively^33–35^. It is generally known that NO can be effectively reduced at Cu sites. However, the formation of NH_4_NO_3_ impedes NO access to the active Cu sites. Instead, NO reacts with NH_4_NO_3_ at BASs to form N_2_ and NO_2_ through reaction (3) (TPSR shown in Supplementary Fig. 10). Furthermore, the NO and NO_2_ conversion levels over Cu2.6-SSZ-13 and Cu0.4-SSZ-13 under SSCR, FSCR and NO_2_-SCR conditions are separately depicted in Supplementary Fig. 11. For Cu2.6-SSZ-13 sample, the NO conversion under SSCR conditions was remarkably higher than that under FSCR conditions, which indicated that the SSCR reaction pathway was significantly inhibited under FSCR conditions. We ascribed the low NO conversion to the reaction with NH_4_NO_3_ (i.e., FSCR reaction) and the extra NO_2_ conversion to the reaction between NO_2_ and NH_3_. For Cu0.4-SSZ-13 sample, the NO conversion under FSCR conditions was likewise inhibited compared to that under SSCR conditions. Differently, the NO_2_ conversion under FSCR and NO_2_-SCR conditions were relatively higher than the NO conversion under SSCR conditions due to the insufficient Cu active sites for SSCR reaction. As a result, the FSCR rates of NO_*x*_ can also be higher than SSCR rates of NO_*x*_ especially when the Cu-zeolite behaves low NO conversion (low Cu loadings, hydrothermal aging state, etc.), which was observed in previous studies^23,27,36,37^. In another word, the NO conversion was inhibited in the presence of NO_2_, while the effect of NO_2_ on NO_*x*_ conversion was uncertain and relates to NO_2_ conversion under FSCR conditions as well as NO_*x*_ conversion under SSCR conditions.

### Wavelet transform analysis of in situ EXAFS measurements

**Fig. 3:**
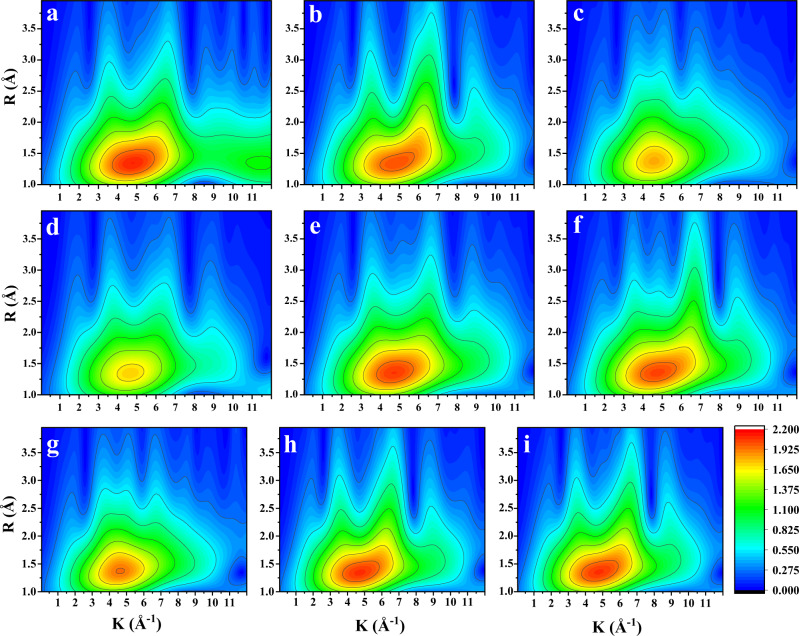
WT plots for EXAFS spectra of Cu-SSZ-13 treated under different conditions at 200 °C. **a** Cu-SSZ-13 pretreated in O_2_/He. **b** NO adsorption. **c** NH_3_ adsorption. **d** NO + NH_3_ adsorption. **e** NO + NH_3_ co-adsorption followed by reaction with O_2_. **f** NO + NH_3_ co-adsorption followed by reaction with NO_2_. **g** SSCR conditions. **h** FSCR conditions. **i** NO_2_-SCR conditions.

Further, we conducted in situ XAFS measurements on Cu-SSZ-13 samples to uncover the valence state and coordination of copper species under different conditions. Wavelet transform (WT) analysis of extended X-ray absorption fine structure (EXAFS) spectra is a powerful technique to resolve overlapping contributions from different neighbor atoms at close distances around the absorber. As shown in Fig. 3a, the pretreated sample shows a distinct first shell peak at (4.5 Å^-1^, 1.3 Å), which is associated with contributions from framework oxygen atoms. This result suggested that the copper species mainly exist as fw-Cu^2+^ species, which have high coordination numbers^28^. For the second shell sphere (R(Å) > 2 Å), two lobes, at (3.5 Å^−1^,2.8 Å) and (6.5 Å^−1^ 3.3 Å), are well-resolved due to the different backscattering properties of various atoms, which strongly depend on the atomic number. The first lobe is assigned to the second-shell oxygen atom due to the low k value of oxygen atoms. The latter one is attributed to the signals from the Si or Al atoms of the framework. Although some studies attributed the latter lobe to the Cu-Cu contributions in oxygen-bridged Cu dimers^38,39^, we scarcely observed CuO_*x*_ species in X-ray absorption near edge structure (XANES) and EXAFS profiles (Supplementary Fig. 12) and did not carry out the procedure of introducing O_2_ to NH_3_-treated Cu-SSZ-13 to form oxygen-bridged Cu dimers with four NH_3_ ligands. Therefore, we deduced that the lobe at 6.5 Å^−1^ is primarily derived from the framework Si or Al atoms in the second shell in this work. In fact, the copper species in Cu-SSZ-13 are initially in the solvated state as [Cu(H_2_O)n]^2+^ under ambient conditions, which weakens the interaction between copper species and the zeolite framework^28,40^. High-temperature treatment in O_2_/N_2_ removes the coordinated water molecules and oxidizes copper species to Cu^2+^. As a result, the copper species are in a high valence state and strongly interact with the zeolite framework through electrostatic forces.

After NO adsorption, Cu^2+^ ions are partially reduced, resulting in a slight decrease in the coordination numbers (CNs) of the first shell, denoted by the decrease and weakening of the colored area (Fig. 3b). The lobes resulting from the contributions of the second shell stretched to (3.5 Å^−1^, 3.1 Å) and (6.5 Å^−1^, 3.7 Å), respectively. When the pretreated sample was exposed to an NH_3_ or NO + NH_3_ atmosphere, the signal of the first shell sharply decreased (Fig. 3c, d), suggesting that the CNs of the Cu ions significantly declined due to their reduction. Moreover, the two lobes are not well-resolved in the spectra, indicating a decrease in the scattering from the second shell. This is consistent with the formation of dynamic [Cu(NH_3_)_2_]^+^ species, which is supported by the appearance of feature B in Supplementary Fig. 13a after NH_3_ or NO + NH_3_ adsorption. After oxidation by O_2_ and NO_2_, the CNs of the first shell increased to a level similar to that of the pretreated sample, accompanied by the formation of two well-resolved lobes at the second shell (Fig. 3e, f). This demonstrated that Cu(NH_3_)_2_^+^ species are oxidized into Cu^II^ ions and that the interaction between the Cu^2+^ ions and the zeolite framework is recovered. Besides the scattering by framework Si (or Al), the second lobe at 6.5 Å^−1^ probably resulted from the scattering of the second shell Cu species, since oxygen-bridged Cu dimers are formed after Cu^I^(NH_3_)_2_ oxidation by O_2_^5,38^. Compared with oxidation by O_2_, oxidation by NO_2_ resulted in a higher signal for the lobe at ~6.5 Å^−1^, indicating that more Cu^I^ species are oxidized into Cu^II^ ions (fw-Cu^II^ or NH_3_-solvated Cu^II^ species with high CNs) during the reaction with NO_2_. This phenomenon is consistent with the result reported by Paolucci et al. showing that NO_2_ can oxidize the residual Cu^I^(NH_3_)_2_ species that cannot be oxidized by O_2_. As also reported by Paolucci, the transient oxidation of Cu^I^(NH_3_)_2_ species by NO_2_ is a single-site process without formation of Cu dimers. Therefore, it can be inferred that the presence of NO_2_ probably changed the SCR reaction active sites from dimer Cu to isolated Cu species, which further influence the SSCR reaction. This deduction indicated that most Cu species are bonded with the zeolite framework and that the mobility of Cu species is limited during the process of Cu^I^(NH_3_)_2_ oxidation by NO_2_. Although the transient reaction can reflect the Cu state and coordination during half-cycles, it was deemed more meaningful to identify the Cu species under FSCR reaction conditions.

**Fig. 4:**
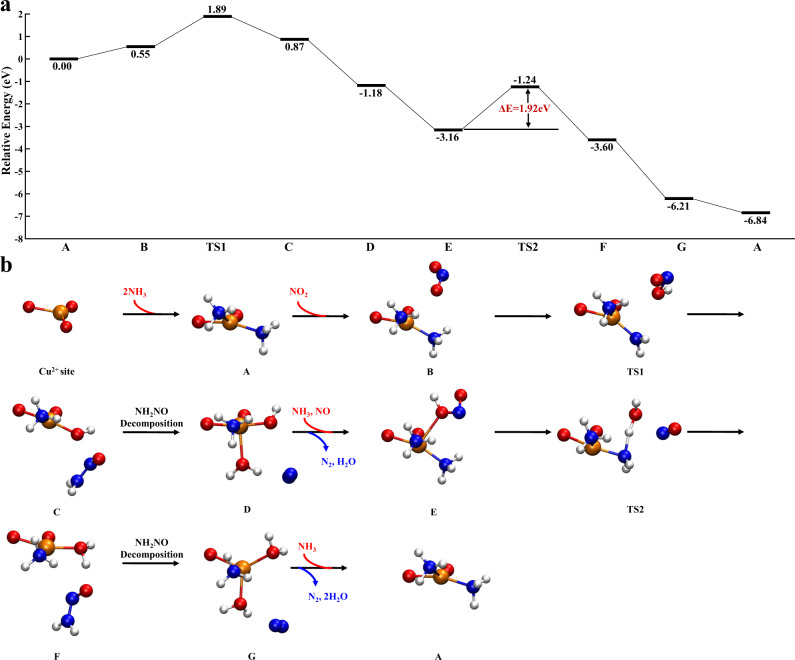
Reaction pathway of the fast SCR cycle at Z_2_Cu^II^ site. **a** Gibbs free energy profile. **b** Optimized geometries of the reactants, transition states (TSs) and products for all elementary steps are presented in the lower panel. Except for the O atoms linked to the Cu^2+^ ion, all other atoms of the zeolite framework are omitted for clarity. Orange, red, blue and white circles denote Cu, O, N and H atoms, respectively.

Figures 4g–i depicts 2D plots of the WT EXAFS spectra under SSCR, FSCR and NO_2_-SCR conditions. Under SSCR conditions, the WT EXAFS spectra resemble the ones in Fig. 3c, d. The first shell peak weakened under SSCR conditions, indicating a decrease in the CNs of Cu species. The absence of the lobes at the second shell suggests the easy mobility of the copper complex due to the NH_3_ solvation effect. In the presence of NO_2_, however, the CNs of the first shell significantly increased, indicating the oxidation of copper species, which was also supported by the results of McEwen et al.^26^. Moreover, two well-resolved lobes at the second shell are observed, suggesting that oxidization leads to the copper species becoming closely coordinated with the zeolite framework, which limits their mobility during the SCR reaction. The WT EXAFS spectra are consistent with the Fourier-transformed (FT) EXAFS results (Supplementary Fig. 14 and Table 1), which are discussed in detail in the Supporting Information. The above results proved the existence of greater amounts of dynamic Cu^I^(NH_3_)_2_ species under SSCR reaction conditions than that under FSCR and NO_2_-SCR reaction conditions. Notably, although we proved the existence of significant framework-bound Cu^II^ species under FSCR conditions, the NH_3_-solvated Cu^II^ species cannot be ruled out by the XAFS experiment. Indeed, the NH_3_-solvated Cu^II^ species existed, as indicated by the observation of NH_3_ desorption from Cu sites in FSCR-TPD profiles (Supplementary Fig. 5), which was consistent with the computed phase diagram reported by Paolucci et al.^28^. Therefore, we next turned to the DFT calculation to investigate the possible FSCR reaction pathways over fw- and NH_3_-solvated Cu^II^ [Cu^2+^ and (Cu^II^OH)^+^] species, BASs and dimer Cu species.

### DFT calculation

We first calculated the FSCR reaction pathway over fw-Cu^II^ species (Fig. 4). The framework-bound Cu^II^ first adsorbs two NH_3_ molecules without separation from the framework, which then interacts with NO_2_ to form Z_2_Cu^II^NH_3_OH and NH_2_NO species (B → C). The Z_2_Cu^II^NH_3_OH further adsorbs an NH_3_ molecule and reacts with NO, resulting in the formation of Z_2_Cu^II^NH_3_, NH_2_NO and H_2_O (E → F), which was predicted to be the rate-determining step of the SCR reaction cycle with a high energy barrier of 1.92 eV. The formed NH_2_NO is easily decomposed into N_2_ and H_2_O through a series of H-migration and isomerization processes (Supplementary Fig. 16)^29^. Last, the gaseous NH_3_ molecules are supplied to regenerate the initial A species.

**Fig. 5:**
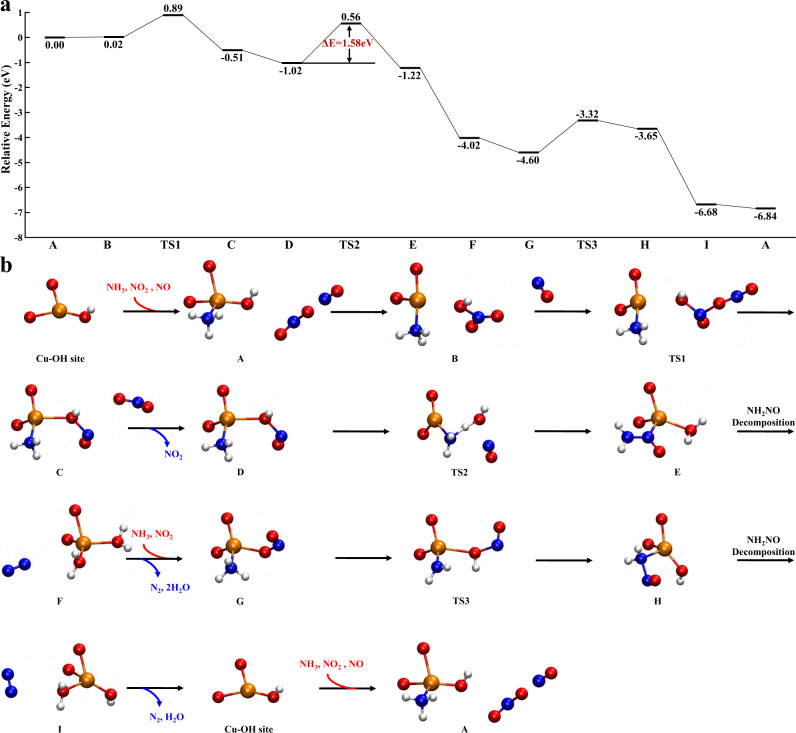
Reaction pathway of the fast SCR cycle at ZCu^II^OH site. **a** Gibbs free energy profile. **b** Optimized geometries of the reactants, TSs and products for all elementary steps are presented in the lower panel. Except for the two O atoms linked to the Cu-OH group, all other atoms of the zeolite framework are omitted for clarity. All legends are the same as those in Fig. 4.

Next, the FSCR reaction pathway over ZCu^II^OH was calculated and depicted in Fig. 5. ZCu^II^OH first adsorbs an NH_3_ molecule to reach a coordinatively saturated state, which interacts with NO_2_ to form an HNO_3_ molecule without any energy barrier. The B species is actually considered to be NH_4_NO_3_ adsorbed on Cu sites. Next, the adsorbed HNO_3_ reacts with NO from the gas phase with an energy barrier of 0.87 eV, resulting in the formation of adsorbed HNO_2_ and the release of an NO_2_ molecule (C → D). Then, the adsorbed HNO_2_ reacts with the NH_3_ ligand to generate NH_2_NO and H_2_O. As the desorption of N_2_ and H_2_O molecules occurs, NH_3_ and NO_2_ are adsorbed at the Cu site and react to generate NH_2_NO and -OH groups. With the decomposition of NH_2_NO into N_2_ and H_2_O, the ZCu^II^OH site is regenerated. The rate-determining step of the FSCR cycle over the ZCu^II^OH site corresponds to the reaction of adsorbed HNO_2_ with an NH_3_ ligand to produce NH_2_NO and H_2_O (E → F), with an energy barrier of 1.58 eV.

In addition, the FSCR reaction pathways over NH_3_-solvated Cu^II^ species [Cu^II^ and (Cu^II^OH)^+^] were also calculated and presented in Supplementary Fig. 17, 18. All the energy barriers were found to be relatively high (1.54 and 1.65 eV). Moreover, we consider the possibility that various NH_3_-solvated Cu^II^ species diffuse into an adjacent cage to form Cu^II^-pairs as shown in Supplementary Fig. S15. As expected, the formation of Cu^II^ pairs from Cu^II^(NH_3_)_4_ and Cu^II^NO_2_(NH_3_)_3_ is both thermodynamically and kinetically inhibited due to the steric effect as well as strong interaction with zeolite framework. However, it should be noted that Cu^II^OH(NH_3_)_3_ is different from the other forms since binuclear Cu^II^OH(NH_3_)_3_ in one cage is thermodynamically more stable than the isolated configuration. Villamaina et al. validated the formation of Cu^II^(OH)(NH_3_)_*x*_ dimeric complexes in the oxidation atmosphere through CO + NH_3_ titration experiment^41^. Hu et al. proposed that Cu^II^(OH)(NH_3_), which is structurally similar to Cu^I^(NH_3_)_2_^+^ that has one charge and two ligands, acts as inter-cage transportation medium^6^. The Z2Cu^II^ species can transform ZCu^II^(OH) by NH_3_-assisted hydrolysis to achieve the Cu pairing^42^. However, the regeneration of Cu^II^(OH) in dimeric form showed a high energy barrier of 1.58 eV (A → B in Supplementary Fig. S19), suggesting that the dimeric Cu^II^(OH) species were not highly active in the FSCR reaction.

**Fig. 6:**
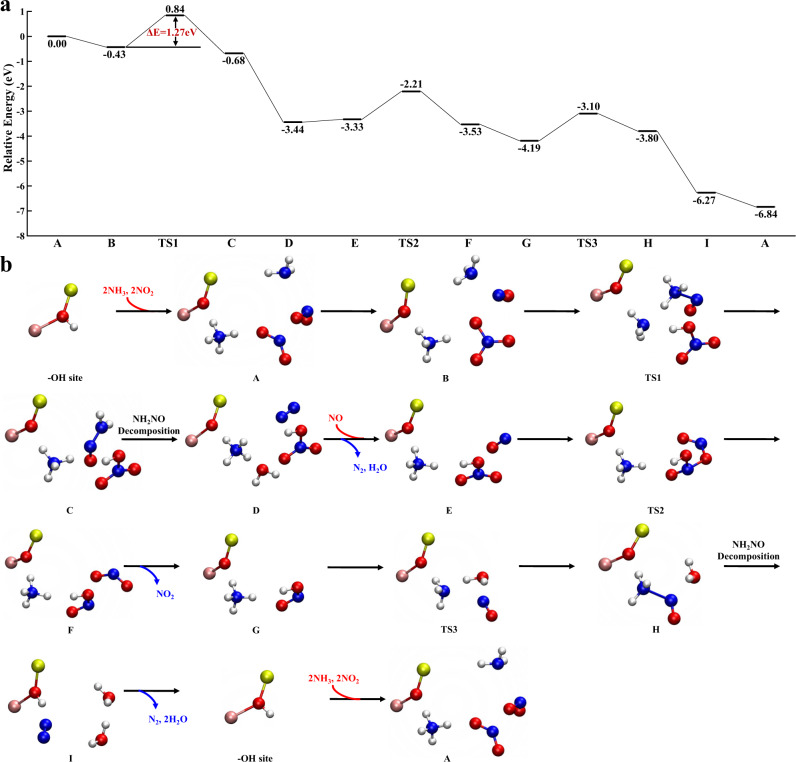
Reaction pathway of the fast SCR cycle at BAS. **a** Gibbs free energy profile. **b** Optimized geometries of the reactants, TSs and products for all elementary steps. Except for the Si and Al atoms linked to the OH group, all other atoms of the zeolite framework are omitted for clarity. Yellow and pink circles denote Si and Al atoms, respectively. All other legends are the same as those in Fig. 4.

The FSCR reaction pathway at BASs is displayed in Fig. 6. NH_3_ is adsorbed on the BASs to form NH_4_^+^ species. Two NO_2_ molecules interact with the NH_4_^+^ species to form NH_4_NO_3_ species and release an NO molecule without any energy barrier (A → B). The release of NO was also observed in our previous studies during NO_2_ adsorption on H-SSZ-13 zeolite^43^. Then, NO interacts with an NH_3_ from the gas phase to form an NH_3_ ∙ ∙ ∙ NO complex, which further reacts with NH_4_NO_3_ to form NH_4_^+^, HNO_3_, and NH_2_NO via an H-migration process (B → C). NH_2_NO is decomposed into N_2_ and H_2_O. Subsequently, HNO_3_ reacts with NO from the gas phase, resulting in the formation of HNO_2_ and the release of an NO_2_ molecule (E → F). Reaction between HNO_2_ and NH_4_^+^ species leads to the formation of an NH_3_ ∙ ∙ ∙ NO complex and an H_2_O molecule. The NH_3_ ∙ ∙ ∙ NO complex transfers an H atom to regenerate the BAS and changes into NH_2_NO, which then decomposes into N_2_ and H_2_O. The whole catalytic cycle is completed. The overall energy barrier of the FSCR over the Brønsted acid site is 1.27 eV, which corresponds to the reaction between NH_4_NO_3_ and the NH_3_ ∙ ∙ ∙ NO complex, much lower than that over various Cu sites. The DFT-calculated results indicate that the FSCR process in the SSZ-13 zeolite system tends to occur at BASs.

In summary, by combining the analysis of in situ spectroscopic measurements with DFT calculations, we found that NO_2_ leads to the deep oxidation of copper species as Cu^II^ species (fw-Cu^II^ and NH_3_-solvated Cu^II^ with high CNs), which significantly inhibits the mobility of Cu sites. As a result, the FSCR reaction occurs primarily at the BASs even though it has a higher energy barrier (1.27 eV) than the locally homogeneous SSCR reaction at dynamic sites (about 1.0 eV). This work reveals the origin of the abnormal NH_3_-SCR behavior over the commercial Cu-SSZ-13 catalyst in the presence of NO_2_.

## Methods

### Sample preparation

The initial Cu-SSZ-13 zeolite was in situ synthesized by a one-pot method^15^. The ratio of Na_2_O/Al_2_O_3_/H_2_O/SiO_2_/Cu-TEPA was 3.5/1.0/200/25/3 and the crystallization of the zeolite was performed at 120 °C for 5 days. Due to the excess Cu in the initial product, aftertreatments were required to optimize the Cu contents and distribution. In detail, the as-synthesized Cu-SSZ-13 was post-treated with 0.1 mol/L HNO_3_ at 80 °C for 12 h to remove CuO_*x*_ species. After calcination at 600 °C, the sample was stirred in NH_4_NO_3_ solution (0.01~0.2 mol/L) at 40 °C for the second post-treatment process, followed by filtration, washing, drying and calcination at 600 °C. The obtained Cu-SSZ-13 catalysts were Al-rich zeolites with Si/Al of ~5 and various Cu loadings from 0.4 to 3.8 wt.% (Supplementary Table 2).

### Catalyst evaluation

The standard SCR (SSCR), fast SCR (FSCR) and slow SCR (NO_2_-SCR) reactions were carried out in a fixed-bed flow reactor system with an online Nicolet Is50 spectrometer, which was used to detect the concentrations of reactants and products. The SSCR conditions included 500 ppm NO and 500 ppm NH_3_; the FSCR conditions included 250 ppm NO, 250 ppm NO_2_ and 500 ppm NH_3_; the NO_2_-SCR conditions included 300 ppm NO_2_ and 500 ppm NH_3_. All the conditions included 3.5% H_2_O, 5%O_2_ and N_2_ balance. The total flow rate was 500 mL/min. The NO_*x*_ (NO and NO_2_) conversion was calculated at steady state:

5 $${NO}\,{conversion}=\left(1-\frac{{\left[{NO}\right]}_{{out}}}{{\left[{NO}\right]}_{{in}}}\right)\times 100\%$$

6 $${{NO}}_{2}\,{conversion}=\left(1-\frac{{\left[{{NO}}_{2}\right]}_{{out}}}{{\left[{{NO}}_{2}\right]}_{{in}}}\right)\times 100\%$$

7 $${{NO}}_{x}\,{conversion}=\left(1-\frac{{\left[{{{{{\rm{NO}}}}}}\right]}_{{{{{{\rm{out}}}}}}}+{\left[{{{{{{\rm{NO}}}}}}}_{2}\right]}_{{{{{{\rm{out}}}}}}}}{{\left[{{{{{\rm{NO}}}}}}\right]}_{{{{{{\rm{in}}}}}}}+{\left[{{{{{{\rm{NO}}}}}}}_{2}\right]}_{{{{{{\rm{in}}}}}}}}\right)\times 100\%$$

To conduct the kinetic studies, the gas hourly space velocity (GHSV) was controlled by adjusting the catalyst weight. The GHSV of SSCR, FSCR and NO_2_-SCR were about ~800,000 h^−1^, ~1,000,0000 h^−1^ and ~2,000,000 h^−1^, respectively. The reaction rates (r) in this study were normalized by catalyst weight based on Eq. (8). The activation energies (Ea) were calculated by the Arrhenius Eq. (9).

8 $$r=\frac{{F}_{{{NO}}_{x}}\bullet {X}_{{{NO}}_{x}}}{{W}_{{cat}}}$$

9 $$r={\left[{{NO}}_{x}\right]}_{0}A{e}^{\left(-\frac{{Ea}}{{RT}}\right)}$$

where F_*NOx*_ represents the NO_*x*_ flow rate (mol/s), X_*NOx*_ represents the NO_*x*_ conversion, W_cat_ is the mass of the catalyst (g), and [NO_*x*_]_0_ is the inlet concentration of NO_*x*_. NOx represents NO, NO_2_ or a mixture of both.

### Characterization

The elemental composition of the catalysts was measured by inductively coupled plasma atomic emission spectroscopy (ICP-AES). N_2_ adsorption-desorption analysis of the samples was conducted on a Micromeritics ASAP 2020 instrument. The acid site distribution and contents were measured by NH_3_ temperature-programmed desorption (NH_3_-TPD) using the NH_3_-SCR activity measurement instrument described above. Samples of about 30 mg were used and pretreated in 10% O_2_/N_2_ at 500 °C for 30 min before cooling down to 120 °C. Then, the gas was changed to 500 ppm NH_3_/N_2_ for 60 min, followed by N_2_ purging for 60 min. Finally, the temperature was raised to 700 °C at a rate of 10 °C/min.

The in situ X-ray absorption fine structure (in situ XAFS) experiments were performed on the 1W1B beamline of Beijing Synchrotron Radiation Facility (BSRF). The absorption data from −200 eV to 800 eV of the Cu K-edge (8979 eV) were collected. The sample was first pretreated in O_2_/He at 500 °C for 30 min before decreasing the temperature to 200 °C, after which the Pre. spectra were collected. Then, the sample was exposed to 500 ppm NH_3_/He, 500 ppm NO/He and 500 ppm NH_3_/He + 500 ppm NO/He for 60 min, respectively, and spectra were collected. After reduction by (NO+NH_3_)/He, the sample was exposed to 5% O_2_/N_2_ and 500 ppm NO_2_/N_2_ for 60 min, respectively, to obtain the absorption data for the oxidized sample. Moreover, the in situ absorption data were collected after the pretreated samples were exposed to SSCR, FSCR and NO_2_-SCR atmospheres for 60 min. The X-ray absorption near-edge structure (XANES) data were background-corrected and normalized using the Athena module implemented in the IFFEFIT software package^44^. Extended X-ray absorption fine structure (EXAFS) data were analyzed and fitted using Athena and Artemis (3.0 < k < 13.0 Å^−1^). An amplitude reduction factor (S_0_^2^) of 0.85 was used for all the fitted data sets. Wavelet transform (WT) analysis of the EXAFS was performed to precisely investigate the local coordination environment of copper species.

### Computational details

Spin-polarized periodic DFT calculations were carried out with the Vienna ab initio simulation package (VASP)^45^ The Perdew−Burke−Ernzerhof (PBE) generalized gradient approximation was adopted with the van der Waals correction proposed by Grimme (i.e., DFT-D3 method)^46^. The Kohn-Sham orbitals were expanded with a plane-wave basis set with a cutoff energy of 500 eV, and the plane augmented wave (PAW) method was used to describe the interaction between the valence electrons and the cores^47^. The DFT + U method was applied to Cu 3d states with U_eff_ = 6.0 eV to describe the on-site Coulomb interactions^29,48^. During geometrical optimization, the self-consistent-field electronic energies were converged to 1 × 10^−5^ eV and all other atoms were fully relaxed until the maximum force on the atoms was less than 2 × 10^−2^ eV/Å. The Brillouin zone was sampled with a Monkhorst-Pack k-point grid of 1 × 2 × 2. The Gaussian smearing method was utilized, with a smearing width of 0.2 eV. The transition states of elementary steps were located using the climbing image nudged elastic band (CI-NEB) method with several intermediate images between initial and final states^49,50^. Thermodynamic data were processed with the VASPKIT code^51^ and the Gibbs free energies were calculated at 200 °C. The SSZ-13 zeolite structure was modelled using two rhombohedral unit cells (24 tetrahedrally coordinated atoms) with size of 18.84 Å × 9.42 Å × 9.42 Å (Supplementary Fig. 20). One Si atom was replaced by one Al atom in each double 6-membered ring, resulting in a model with a Si/Al ratio of 11. One H atom was introduced onto one of the O atoms connected with each Al atom to keep the structure charge-neutral. Based on previous studies^4,29^, the present computational settings and models were reliable for investigating the NH_3_-SCR mechanism over Cu-SSZ-13 zeolites.

## Supplementary information

Supplementary Information

Peer Review File

## Data Availability

All data generated and analyzed in this study are provided in the Article and Supplementary Information, and are also available from the corresponding authors upon reasonable request.
