# Supplementary material for: Strikingly distinctive NH3-SCR behavior over Cu-SSZ-13 in the presence of NO2
Source: Nat Commun. 2022 Aug 8;13:4606. doi: 10.1038/s41467-022-32136-z (PMC9360435; doi:10.1038/s41467-022-32136-z)
Supplement: Supplementary file 1 — Supplementary Information [file 41467_2022_32136_MOESM1_ESM.pdf]

SUPPLEMENTARY MATERIAL

Strikingly Distinctive NH_3-SCR Behavior over Cu-SSZ-13 in the Presence of NO_2

Yulong Shan[1], Guangzhi He[1,*], Jinpeng Du[2], Yu Sun[1,4], Zhongqi Liu[1,4], Yu Fu[1,4], Fudong Liu[3], Xiaoyan Shi[1,4], Yunbo Yu[1,2,4], Hong He[1,2,4,*]

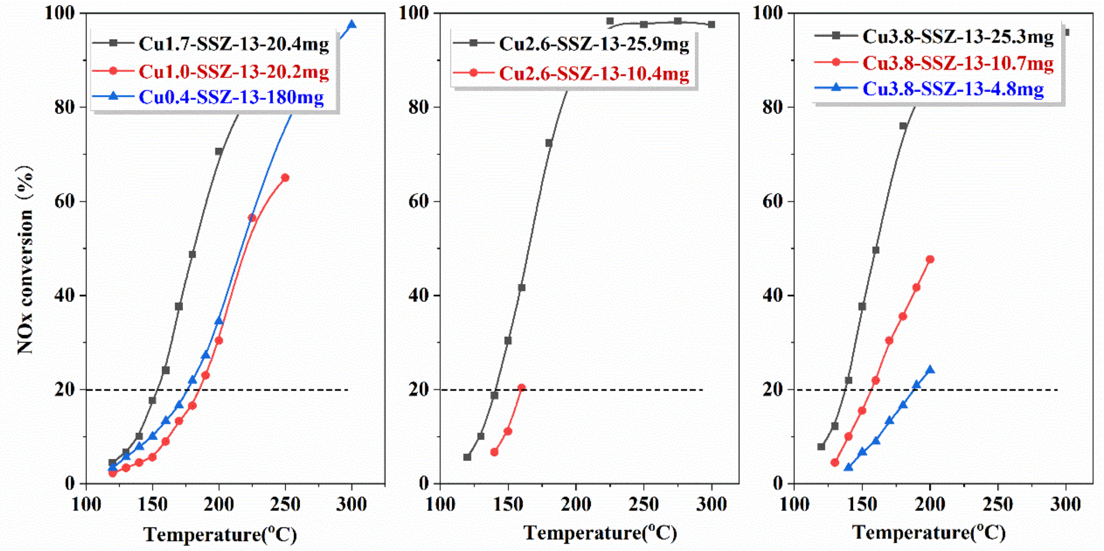

Supplementary Fig. 1 The SSCR behaviors of Cu-SSZ-13 with various Cu loading. NO_x conversion over Cu-SSZ-13 with different Cu loadings under SSCR conditions. SSCR conditions: [NO]=500 ppm, [NH_3]=500 ppm, [O_2]=5 vol.% [H_2O]=3.5 vol.%, Total flow=500 mL/min.

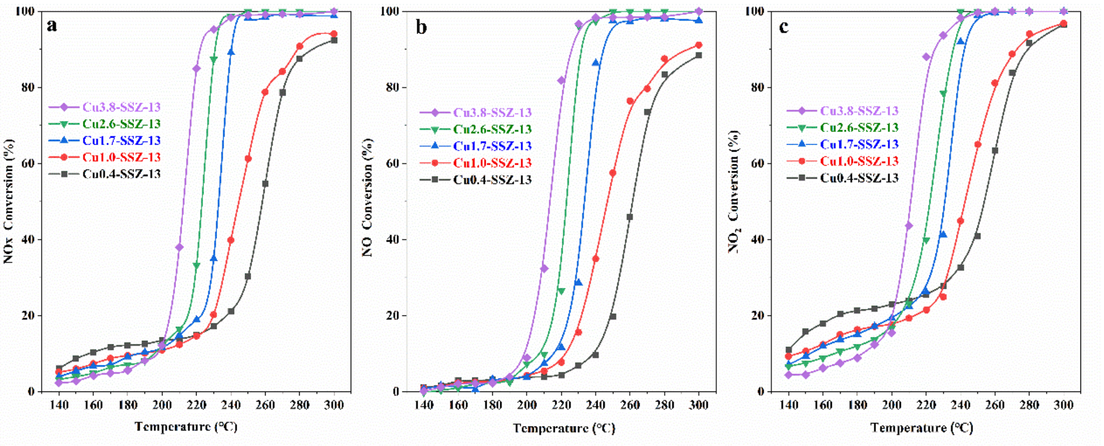

Supplementary Fig. 2 The FSCR behaviors of Cu-SSZ-13 with various Cu loading. (**a**) NO_x conversion, (**b**) separate NO conversion and (**c**) separate NO_2 conversion of Cu-SSZ-13 with different Cu loadings under FSCR conditions. [NO]=[NO_2]=250 ppm, [NH_3]=500 ppm, [O_2]=5 vol.% [H_2O]=3.5 vol.%. GHSV = 800,000 h^{-1}.

Generally, the NO_x conversion was significantly inhibited at low temperatures (< 200 °C, Supplementary Fig. 3a) due to the formation of NH_4NO_3, which has been reported in previous studies and verified in this study by the observation of N_2O emission in an FSCR-TPD experiment (Supplementary Fig. 5b).[1,2] The inhibition

effect results from the negligible degree of NO reduction at low temperatures (Supplementary Fig. 1b), while NO_2 conversion made the primary contribution to the NO_x reduction (Supplementary Fig. 1c). This is because NO primarily reacted at active Cu sites, while the formed NH_4NO_3 made the Cu species inaccessible to NO. Moreover, large amounts of NH_4NO_3 are easily formed on the Al-rich zeolite that was used in this work. In addition, it should be noted that the NO_2 conversion decreased with increasing Cu loading. As the temperature increased to the decomposition temperature of NH_4NO_3, the deNO_x efficiency was rapidly elevated and the both the NO and NO_2 conversion levels increased with the increase in Cu loading.

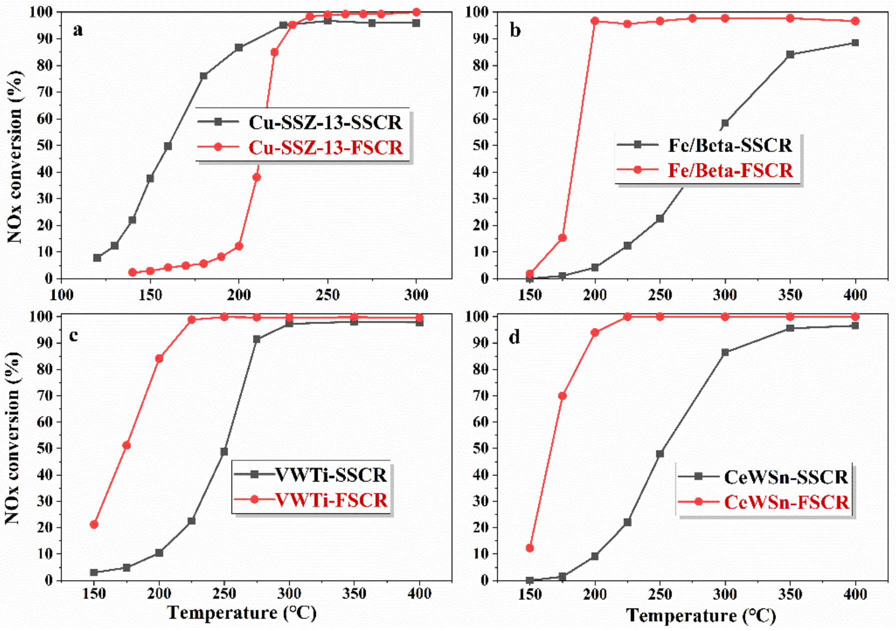

Supplementary Fig. 3 Comparison of SSCR and FSCR behaviors over different catalysts. (**a**) NO_x conversion of Cu-SSZ-13, (**b**) NO_x conversion of Fe/Beta, (**c**) NO_x conversion of $VWTiO_x$ and (**d**) NO_x conversion of $CeWSnO_x$ catalysts under FSCR and SSCR conditions. FSCR conditions: [NO]=[NO_2]=250 ppm, [NH_3]=500 ppm, [O_2]=5 vol.% [H_2O]=3.5 vol.%. SSCR conditions: [NO]=500 ppm, [NH_3]=500 ppm, [O_2]=5 vol.% [H_2O]=3.5 vol.%. The GHSVs were 800,000, 400.000, 200,000 and 400,000 h^{-1}, respectively. The Cu and Fe contents of Cu-SSZ-13 and Fe/Beta were 3.8 and 2.0 wt.%, respectively. The molar ratio of Ce:W:Sn in the $CeWSnO_x$ catalyst was 1:0.2:2. The V and W contents for $VWTiO_x$ were 0.5 and 7.5 wt.%, respectively, and the support was anatase TiO_2.

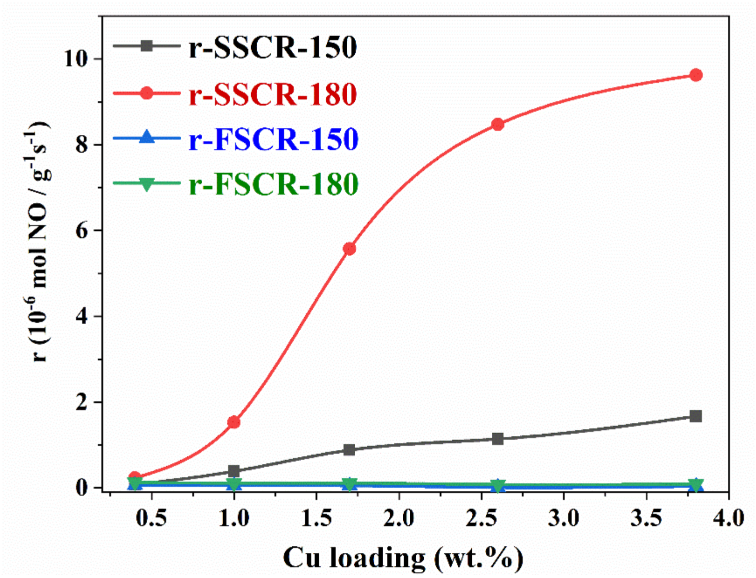

Supplementary Fig. 4 Comparison of NO reaction rates under SSCR and FSCR conditions. NO rates as a function of Cu loading over Cu-SSZ-13 catalysts under SSCR and FSCR conditions at 150 and 180℃.

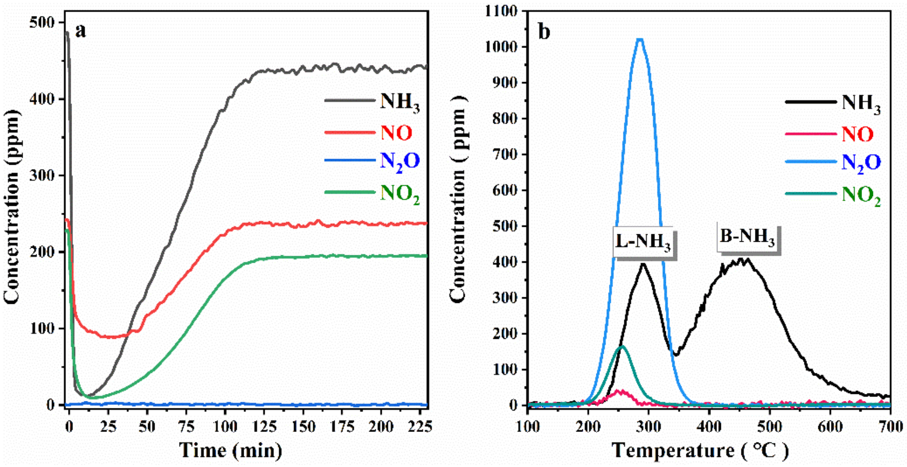

Supplementary Fig. 5 FSCR reaction and TPD behaviors over Cu2.6-SSZ-13. NH_3, NO, NO_2 and N_2O concentrations (**a**) during FSCR reaction process and (**b**) during TPD process of the sample treated by FSCR atmosphere.

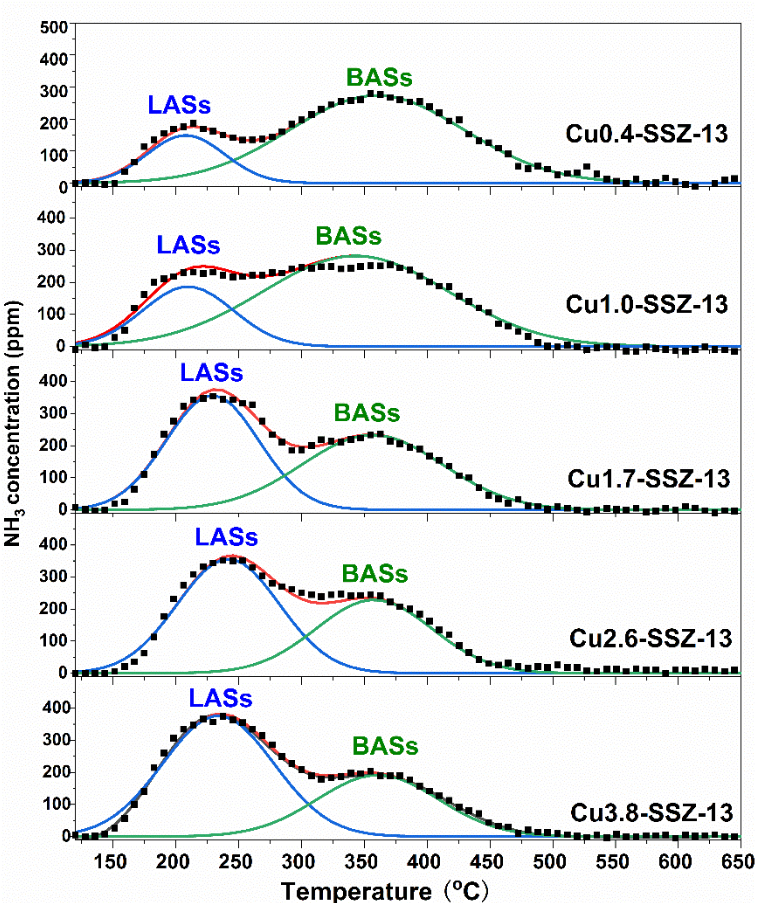

Supplementary Fig. 6 Acid site distribution of Cu-SSZ-13 catalysts. NH_3-TPD profiles of Cu-SSZ-13 with different Cu loadings

Supplementary Fig. 6 shows the NH_3-TPD profiles of Cu-SSZ-13 with various Cu loadings. There are two primary NH_3 desorption peaks at ~ 230 and ~360 °C, which are ascribed to NH_3 adsorbed on Lewis acid sites (LASs, primarily Cu sites) and Brønsted acid sites (BASs, primarily -Si-OH-Al-), respectively.[3,4] The total amount of NH_3 desorption from LASs and BASs was calculated and shown in Supplementary Table 2. The number of BASs increased, while LASs decreased with decreasing Cu loading. This demonstrated that active Cu^{2+} sites occupied the BASs in zeolite, converting BASs into LASs, which is consistent with previous studies.[3,5] Therefore, the LASs and BASs are in inverse proportion. As consequence, the NO_2 reaction rates decreased with increasing Cu loading but increased with the amounts of BASs.

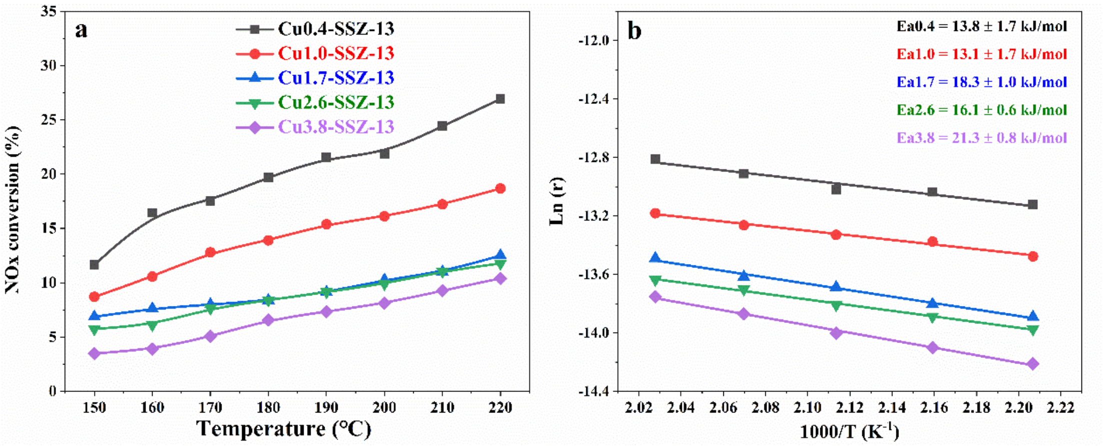

Supplementary Fig. 7 NH_3-SCR performance of Cu-SSZ-13 with different Cu loadings under NO_2-SCR conditions. a The NOx conversion over Cu-SSZ-13 with different Cu loading under NO_2-SCR conditions. **b** The activation energies of NO_2-SCR reaction over Cu-SSZ-13 with different Cu loading. Conditions: $[NO_2]$=350 ppm, $[NH_3]$=500 ppm, $[O_2]$=5 vol.% $[H_2O]$=3.5 vol.%. GHSV = 2,400,000 h^{-1}.

During the NO_2-SCR reaction, the NO_2 conversion decreased markedly with increasing Cu loading, indicating that NO_2 rarely reacted at Cu sites. The activation energies of the NO_2-SCR reaction are between 16-22 kJ/mol, indicating facile reaction between NO_2 and NH_3 over Cu-SSZ-13. The reaction between NO_2 and NH_3 always resulted in NH_4NO_3 formation through the following reaction:[6,7]

$$2NO_2 + 2NH_3 = NH_4NO_3 + N_2 + H_2O \quad (1)$$

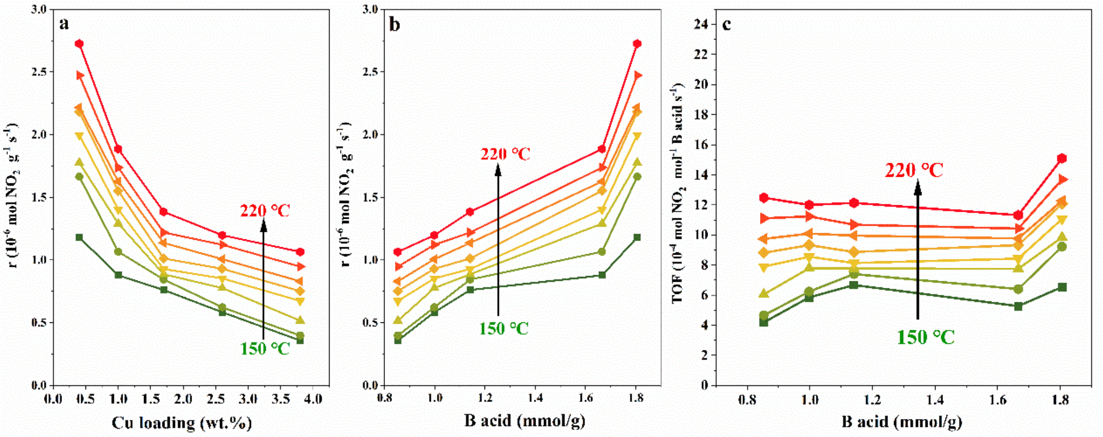

Supplementary Fig. 8 The correlation between NO_2 reaction rates and active sites. NO_2 reaction rates as a function of (**a**) Cu loading and (**b**) Brønsted acid sites under NO_2-SCR conditions. **c** NO_2 turnover frequency (TOF) as a function of Brønsted acid sites under NO_2-SCR conditions.

Under NO_2-SCR conditions, the NO_2 reaction rate decreased with the increase in Cu loading (Supplementary Fig. 8a) while increasing with the increase in BASs (Supplementary Fig. 8b), indicating that the BASs rather than the Cu^{2+} sites participated in the reduction of NO_2. Moreover, the TOF of NO_2 as a function of the amount of BASs is approximately constant (Supplementary Fig. 8c), suggesting a single-site catalysis process.

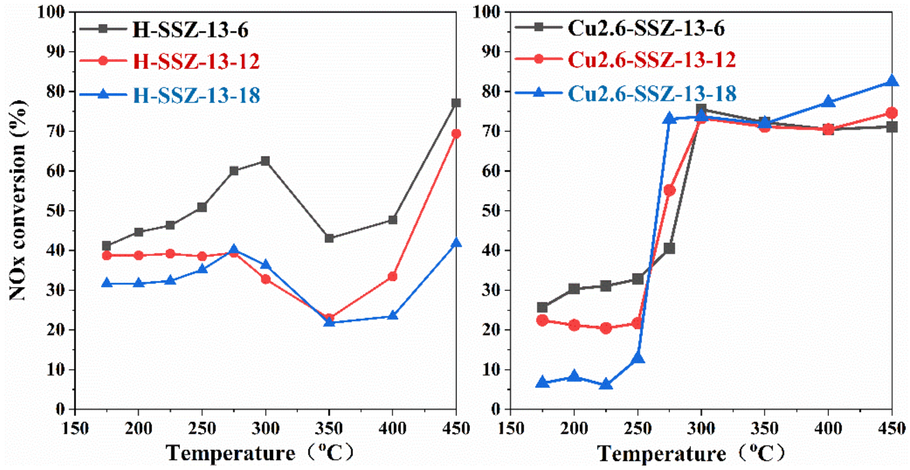

Supplementary Fig. 9 The effects of Si/Al on the NO_2-SCR reaction. NO_x conversion over H-SSZ-13 and $Cu_{2.6}$-SSZ-13 with Si/Al of 6, 12 and 18 under NO_2-SCR conditions.

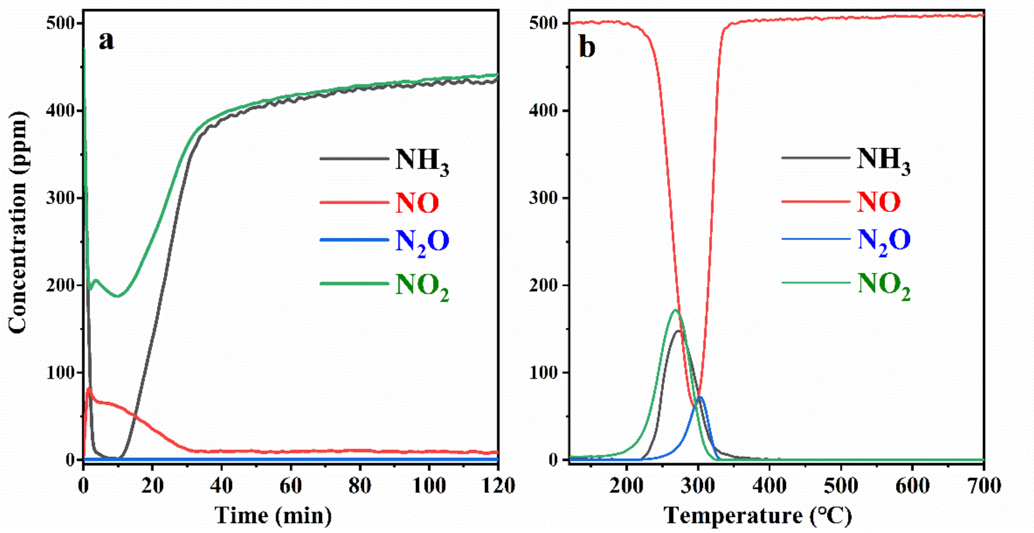

Supplementary Fig. 10 The NH_4NO_3 formation and decomposition. a NO_2 and NH_3 co-adsorption over $Cu_{2.6}$-SSZ-13 as a function of time at 120°C. **b** NO-TPSR reaction over NO_2 and NH_3 co-treated $Cu_{2.6}$-SSZ-13.

The appearance of a weak NO desorption peak indicated the disproportionation of NO_2 during the NO_2 and NH_3 co-adsorption process. Actually, NO_2 disproportionation occurs on the BASs to form nitrates and adsorbed NO^+, which then react with NH_3 to form NH_4NO_3 and NH_2NO, respectively. The consumption of NO, accompanied with the appearance of NO_2, indicated the reaction between NO and NH_4NO_3 (reaction 4). NO^+ could also react with NH_3 to form NH_2NO (reaction 5). NH_2NO then easily decomposes to N_2 and H_2O (reaction 6). This process was also observed in previous studies.[6,8-11]

$$2NO_2 + ZH \rightarrow ZNO + HNO_3 \quad (2)$$

$$HNO_3 + NH_3 \rightarrow NH_4NO_3 \quad (3)$$

$$NO + NH_4NO_3 \rightarrow N_2 + 2H_2O + NO_2 \quad (4)$$

$$ZNO + NH_3 \rightarrow ZHNONH_2 \quad (5)$$

$$ZHNONH_2 \rightarrow ZH + N_2 + H_2O \quad (6)$$

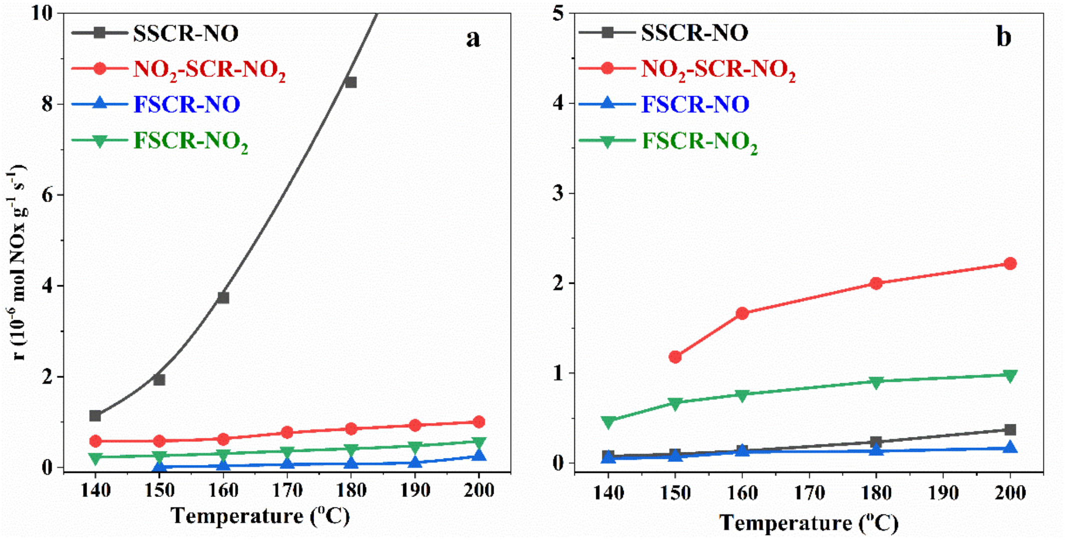

Supplementary Fig. 11 NOx reaction rates under various reaction conditions over Cu-SSZ-13. NO and NO_2 reaction rates over (**a**) Cu2.6-SSZ-13 and (**b**) Cu0.4-SSZ-13 catalysts under SSCR, FSCR and NO_2-SCR conditions.

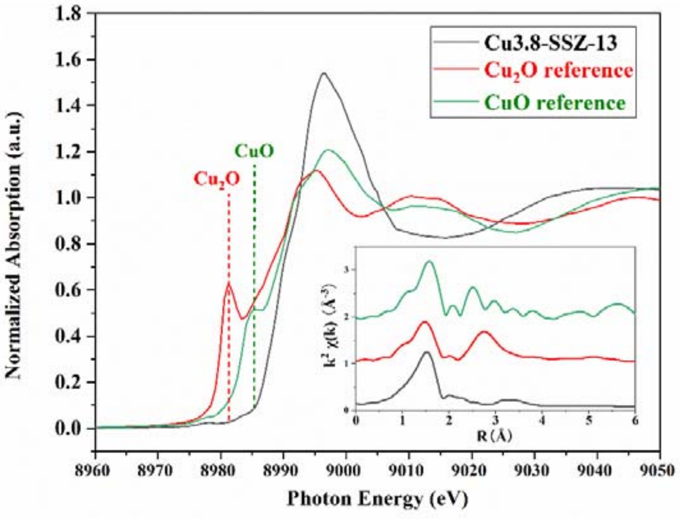

Supplementary Fig. 12 Cu K-edge XANES and EXAFS profiles of CuO, Cu_2O and hydrated $Cu_{3.8}$-SSZ-13 samples.

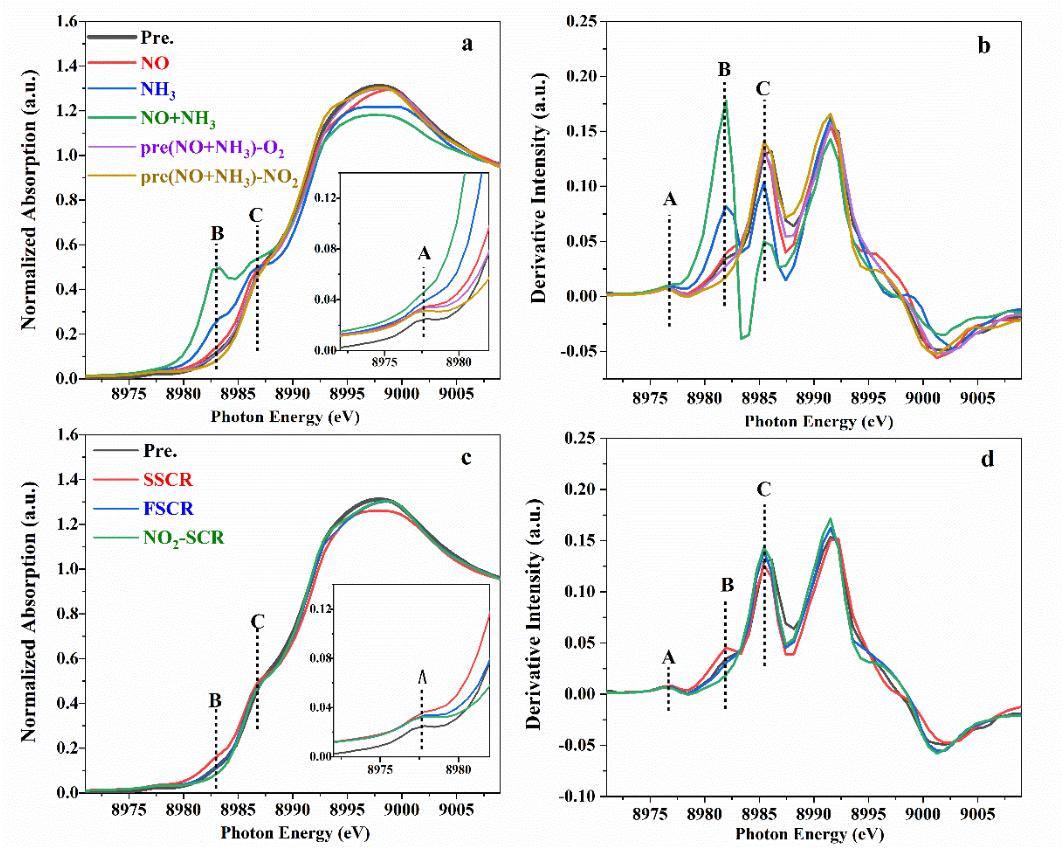

Supplementary Fig. 13 State and coordination of Cu species over Cu-SSZ-13 under different conditions. Cu K-edge XANES (**a**, **c**) and the first derivative spectra (**b**, **d**) of Cu-SSZ-13 treated under different atmospheres. The Cu-SSZ-13 sample was treated by (i) NO adsorption, (ii) NH_3 adsorption, (iii) NO+NH_3 co-adsorption, (iv) NO+NH_3 co-adsorption followed by reaction with O_2, (v) NO+NH_3 co-adsorption

followed by reaction with NO_2, (vi) SSCR reactants, (vii) FSCR reactants and (viii) NO_2-SCR reactants at 200 °C. The sample Cu-SSZ-13 was pretreated in O_2/He at 500 °C for 30 min before decreasing the temperature to 200 °C, after which the Pre. spectra were collected.

Reduction and oxidation of copper species by SCR reactants

Furthermore, we conducted *in-situ* XAFS experiments on Cu-SSZ-13 samples to investigate the state of copper species under different conditions. Cu-SSZ-13 was characterized by XAFS after different SCR reactant treatments at 200 °C. As shown in Supplementary Fig. 13a, results showed a weak pre-edge peak A at ~8977.5 eV, which was assigned to the 1s → 3d transition of d^9 Cu^{2+} ions.[12,13] The B and C features at ~8983 and ~8987 eV are attributed to the 1s → 4p transitions of linearly coordinated Cu^+ ions and fourfold coordinated Cu^{2+} ions, respectively.[14,15] These three features were also clearly observed in the first derivative form of the XANES spectra (Supplementary Fig. 13b). Compared with the pretreated Cu-SSZ-13, both NO and NH_3 induced the partial reduction of Cu^{2+} to Cu^+, indicating that both NO and NH_3 can be activated by Cu^{2+} species through transferring electrons to Cu^{2+}. Moreover, Cu^{2+} ions were more easily reduced by NH_3 due to the facile formation of linear $[Cu(NH_3)_2]^+$ species.[16,17] This is because, compared with NO, NH_3 molecules interact more strongly with Cu^{2+} sites due to the ligand effect between NH_3 and Cu^{2+}, which has been observed in many studies.[18,19] Moreover, it is noted that more $[Cu^I(NH_3)_2]^+$ species were formed when Cu-SSZ-13 was exposed to the NO+NH_3 mixture, indicating that NO can further assist the reduction of Cu^{2+} species by NH_3. The reduction of Cu^{2+} by a NO+NH_3 mixture is generally thought of as the reduction cycle of the SSCR reaction.[18,20]

$$Z_2Cu^{2+} + 3NH_3 + NO \rightarrow ZCu^+(NH_3)_2 + ZH + N_2 + H_2O \quad (7)$$

(Z represents zeolite framework with negative charge)

However, it should be noted that the feature B is not as high as reported in literatures[17,21], indicating that there were probably some irreducible Cu-oxides in the Cu3.8-SSZ-13 sample. Nevertheless, the amount of CuO_x was expected to be insignificant since no obvious CuO_x species were detected in ex-situ XAFS by

comparing with CuO_x standards. To investigate the reactivity of $[Cu(NH_3)_2]^+$ species in an oxidizing atmosphere, O_2 and NO_2 were introduced separately to oxidize Cu^+ ions after treatment by $NO+NH_3$ mixture. After oxidation by O_2, the peak of $[Cu(NH_3)_2]^+$ sharply decreased, concomitant with the appearance of Cu^{2+} ions at ~8977 and ~8987 eV, similar to the XANES profiles of pretreated Cu-SSZ-13. Nevertheless, a slight amount of $[Cu(NH_3)_2]^+$ species still existed as indicated by peak B, which is identified more clearly in the first derivative form of the XANES spectra (Supplementary Fig. 13b). This observation is consistent with previous studies showing that not all Cu^+ can be completely oxidized even in an O_2 atmosphere.[14,17] Moreover, this phenomenon is more clearly proved by the observation of the further decrease in peak B in Supplementary Fig. 13a and 13b after deeper oxidation by NO_2, which demonstrated that NO_2 can further oxidize the residual $[Cu^I(NH_3)_2]$ species since NO_2 has stronger oxidizing power. The complete disappearance of peak B suggests that all the $[Cu^I(NH_3)_2]$ species are oxidized by NO_2. This phenomenon is consistent with the result reported by Paolucci et al that the oxidation of $Cu^I(NH_3)_2$ by O_2 has a theoretical limit, while NO_2 can fully oxidize the $Cu^I(NH_3)_2$ species. Moreover, it was found that the transient oxidation of $Cu^I(NH_3)_2$ species by NO_2 is a single-site process where the first-order rate constant is independent of Cu site density. However, it was known that dimer Cu species participated in the SSCR reaction. The existence of NO_2 probably changed the active sites of SCR reaction from dimer Cu to isolated Cu species, which further influence the SSCR reaction. Compared with Cu^+ species, Cu^{2+} has more ligands, and NO_2 adsorption to form nitrates on Cu^{2+} species is facile. In addition, the higher valence facilitates the interaction between Cu^{2+} ions and the framework. As a result, the Cu^{2+} ions are likely bound to the framework to form fw-Cu^{2+} (framework fixed Cu^{2+}) species, inhibiting the mobility of the copper species.

State of copper species during SSCR, FSCR and NO_2-SCR reactions

To uncover the state and coordination of Cu species under real SCR conditions, we conducted XAFS measurements and depicted the XANES profiles and their first derivative form for Cu-SSZ-13 under SSCR, FSCR and NO_2-SCR conditions, with

the results shown in Supplementary Fig. 13c and 13d. Under SSCR conditions, both features A and B were clearly observed, indicating the coexistence of large amounts of $[Cu(NH_3)_2]^+$ species and Cu^{2+} ions, which suggests the occurrence of redox between Cu^{2+} and Cu^+ ions under SSCR conditions.[22,23] This was also observed in the WT-EXAFS results (Fig. 3). Under FSCR conditions, however, the XANES profile was more like that of pretreated Cu-SSZ-13, demonstrating that most of the copper species were Cu^{II} ions, although there was still a slight amount of Cu^+ ions. This result suggests that Cu^{II} is the prevalent oxidation state under FSCR conditions, which is consistent with the results reported by McEwen et al.[24] This is disadvantageous to the SSCR reaction or NO reduction under FSCR conditions, since the existence of more Cu^{2+} ions means tight coordination with the zeolite and weak mobility for the active copper species. Under NO_2-SCR conditions, furthermore, almost all of the copper species were in the Cu^{2+} state while few Cu^+ species were observed, as indicated by the presence of peak A and the absence of peak B. This demonstrated that the NO_2-SCR is independent of the redox of copper species or that the rate of oxidation of Cu^+ is much higher than the reduction process, which is consistent with the kinetic results (Fig. 2). The *in-situ* XAFS results show that more Cu^{2+} ions exist under NO_2-SCR conditions than in the pretreaxted Cu-SSZ-13, indicating that NO_2 can oxidize the copper species even though they reacted with NH_3 at Brønsted acid sites. This demonstrated that the dynamic $[Cu(NH_3)_2]^+$ species dominated under SSCR conditions, while fw-Cu^{2+} primarily existed under FSCR and NO_2-SCR conditions.

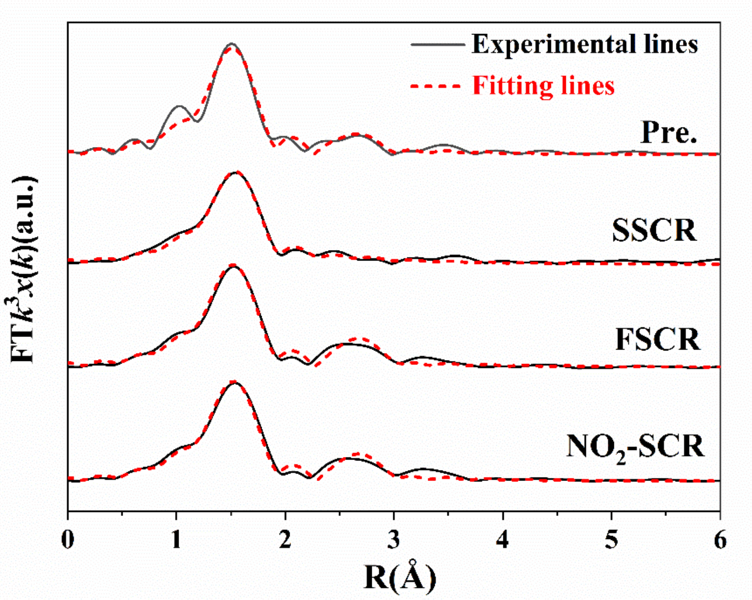

Supplementary Fig. 14 Coordination analysis of Cu species over Cu-SSZ-13 under in-situ reaction conditions. Fourier-transformed (FT) k^3-weighted EXAFS spectra (phase uncorrected) of Cu-SSZ-13 treated under different atmosphere at 200°C.

Supplementary Fig. 14 shows the Fourier-transformed (FT) EXAFS spectra (without phase correction) of Cu-SSZ-13 under various conditions. The first-shell peak is generally attributed to the scattering of O or N atoms, which derived from the zeolite framework O or adsorbed NH_3 in this work.[12,13] The second shell is primarily attributed to the zeolite framework T atoms (Si or Al).[12,25] The appearance of the second shell scattering feature indicated the interaction between copper species and the zeolite framework. On the contrary, the absence of the second shell scattering feature suggests high mobility for the copper species. The parameters resulting from the experimental data and the simulated fittings of EXAFS spectra are listed in Supplementary Table S1.

Compared with the pretreated sample, the Cu-SSZ-13 exposed to SSCR conditions showed decreased intensity for the first-shell peak, with the coordination number (CN) decreasing from 3.7 to 3.5. The decrease in CN indicated the reduction of Cu^{2+} species to $[Cu(NH_3)_2]^+$ species. The absence of the second shell scattering feature for NH_3-treated Cu-SSZ-13 also proved the formation of dynamic

$[Cu(NH_3)_2]^+$ species, which are unconnected with the zeolite framework. However, when NO_2 was involved in the SCR reaction (FSCR or NO_2-SCR), features of the second shell appeared, indicating the scattering of framework atoms (Si or Al). The Cu^{2+} species with high charge dominated in the presence of NO_2 with high oxidation ability. This high charge for the copper species intensified the electrostatic force between the copper species and zeolite framework, leading to the active sites becoming fixed on the zeolite.

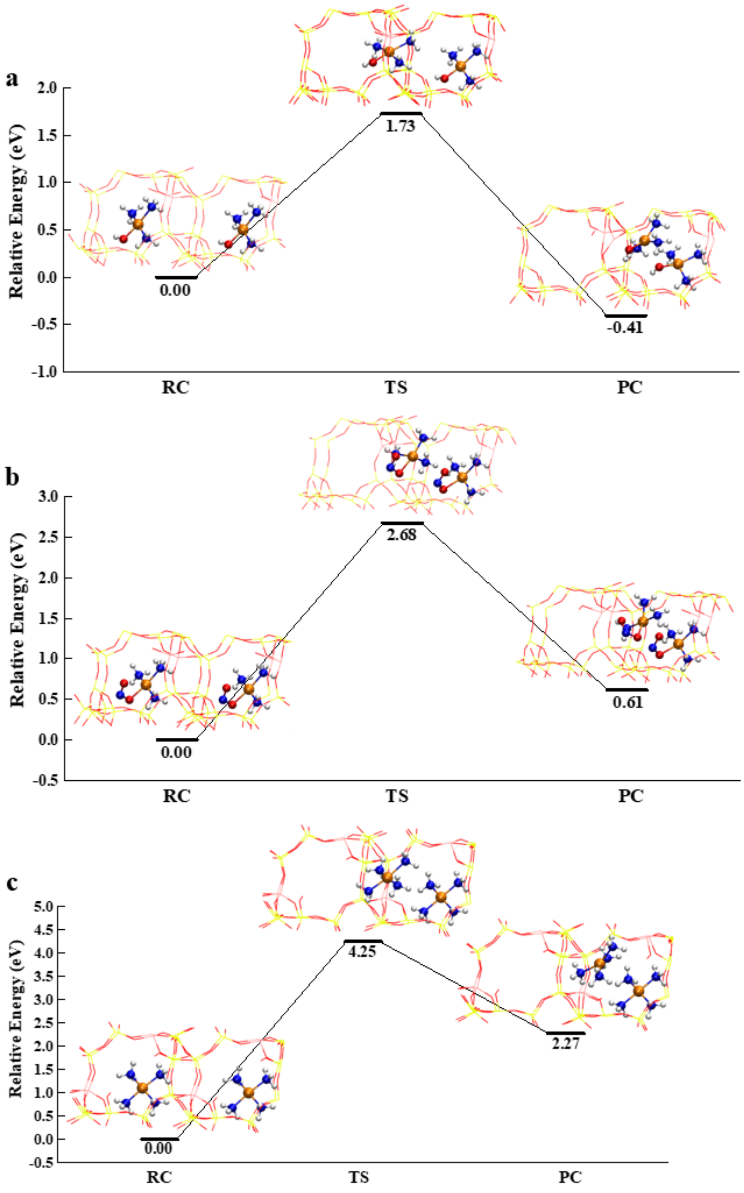

Supplementary Fig. 15 Gibbs free energy profiles for the diffusion of various

NH_3-solvated Cu^{II} species through an 8-MR window into an adjacent cage to form Cu^{II} pairs. a $Cu^{II}OH(NH_3)_3$. **b** $Cu^{II}NO_2(NH_3)_3$. **c** $Cu^{II}(NH_3)_4$. The structures of the reactants (RC), transition states (TS), and products (PC) are presented as insets. The zeolite structure is displayed by thin lines for clarity. Orange, red, blue and white circles denote Cu, O, N and H atoms, respectively.

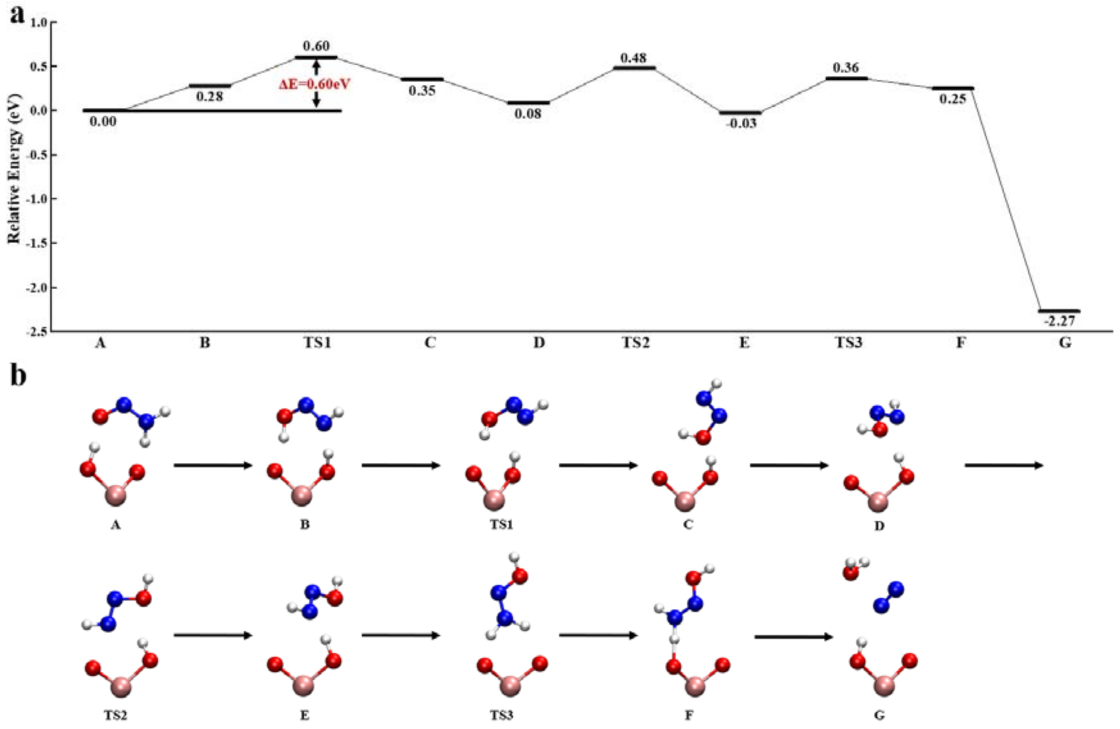

Supplementary Fig. 16 Reaction pathway of NH_2NO decomposition with the assistance of Brønsted acid sites. a Gibbs free energy profile. **b** Optimized geometries of the reactants, transition states (TSs) and products for all elementary steps. Except for the Al-O(H) groups involved in the reaction, all other atoms of the zeolite framework are omitted for clarity. Pink, red, blue, and white circles denote Al, O, N and H atoms, respectively.

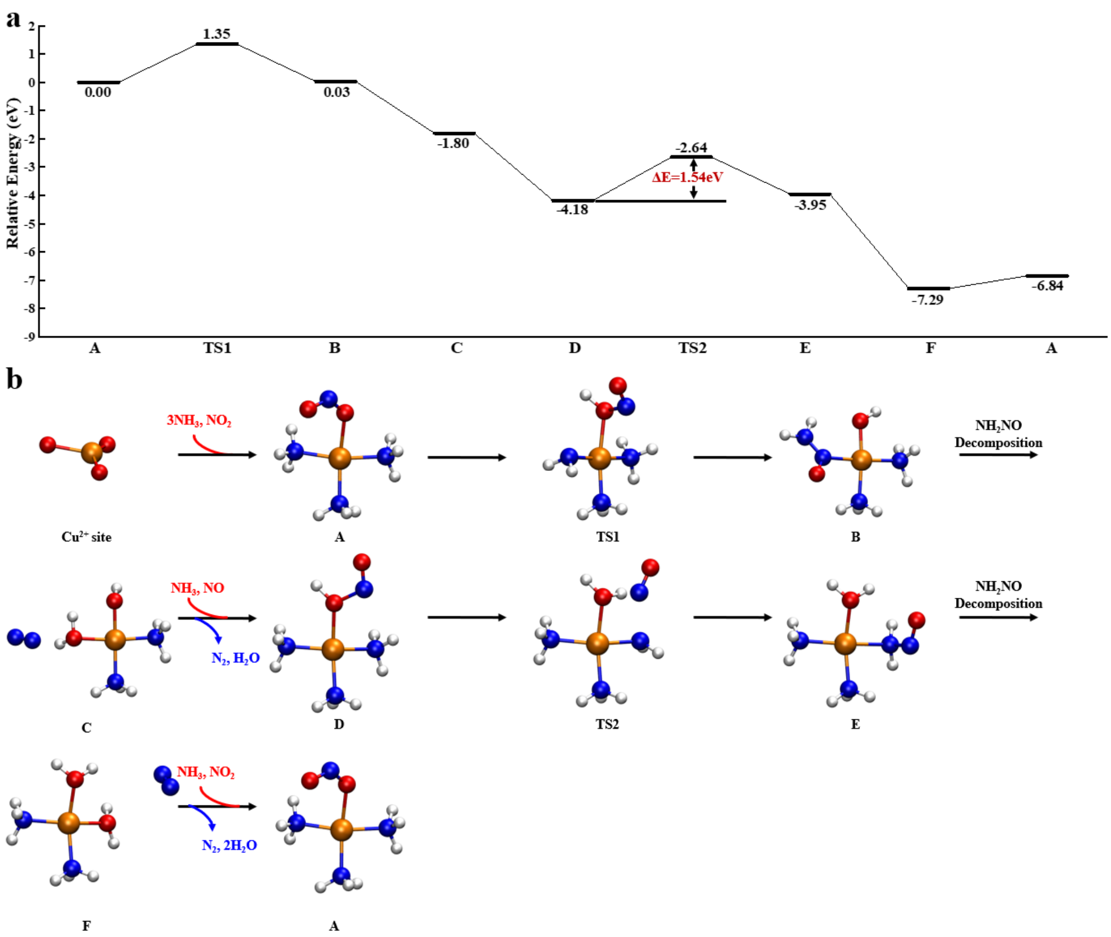

Supplementary Fig. 17. Reaction pathway of the fast SCR cycle at the NH_3-solvated monomeric Cu^{II} site. a Gibbs free energy profile. **b** Optimized geometries of the reactants, TSs and products for all elementary steps. Except for the O atoms linked to the Cu^{2+} ion, all other atoms of the zeolite framework are omitted for clarity. Orange, red, blue and white circles denote Cu, O, N and H atoms, respectively.

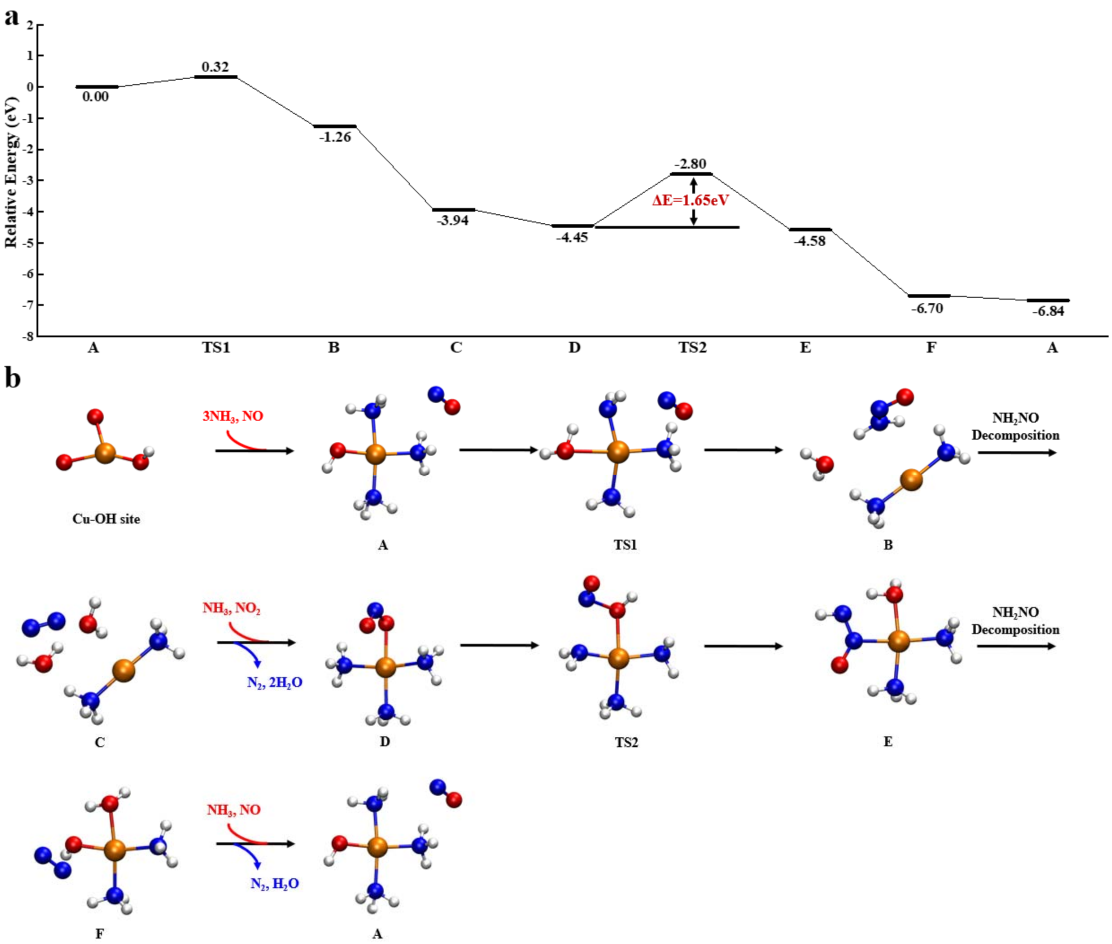

Supplementary Fig. 18. Reaction pathway of the fast SCR cycle at the NH_3-solvated monomeric $Cu^{II}OH$ site. a Gibbs free energy profile. **b** Optimized geometries of the reactants, TSs and products for all elementary steps. Except for the O atoms linked to the Cu-OH group, all other atoms of the zeolite framework are omitted for clarity. All legends are the same as those in Supplementary Fig. 17.

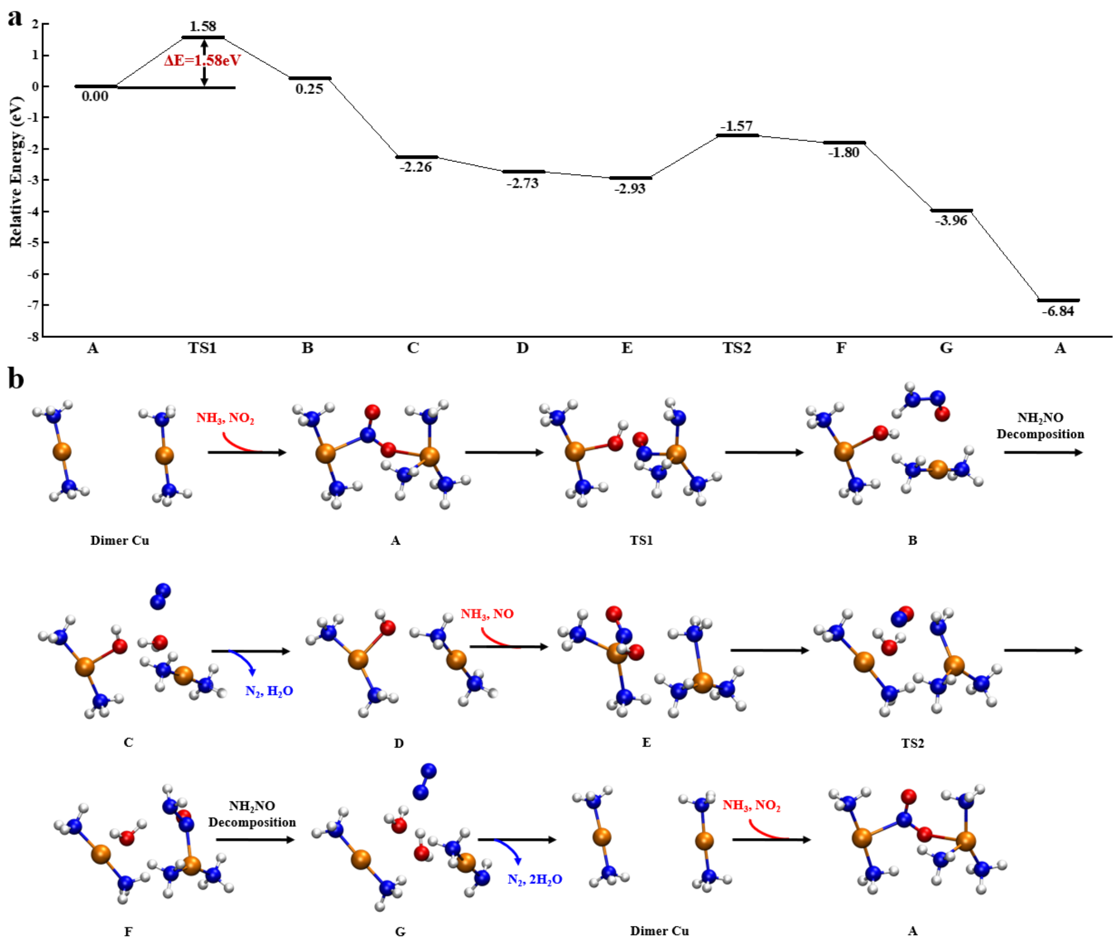

Supplementary Fig. 19. Reaction pathway of the fast SCR cycle at the Cu-dimer site. a Gibbs free energy profile. **b** Optimized geometries of the reactants, TSs and products for all elementary steps. The zeolite framework is omitted for clarity. All legends are the same as those in Supplementary Fig. 17.

The fast SCR pathway with the participation of the Cu dimer was also calculated. First, the adsorption of NO_2 leads to the formation of an NO_2-bridging dimer-Cu (see A in Supplementary Fig. 19). Subsequently, NO_2 is activated and reacts with one adsorbed NH_3 molecule, leading to the formation of $Cu^{2+}OH(NH_3)_2$ and an NH_2NO species (see B in Supplementary Fig. 19). The NH_2NO is then easily decomposed into N_2 and H_2O with a low energy barrier of 0.60 eV (Supplementary Fig. 16). Then, one NO and one NH_3 are adsorbed on $Cu^{2+}OH(NH_3)_2$ and $Cu^{+}(NH_3)_2$, respectively. The adsorbed NH_3 reacts with the -OH group at the other Cu site to generate an H_2O molecule and an -NH_2 species. The -NH_2 species further reacts with the adsorbed NO to form NH_2NO (see F in Supplementary Fig. 19). Accompanied by the

decomposition of NH_2NO into N_2 and H_2O, the Cu-dimer site is regenerated. The highest energy barrier of the FSCR cycle over the Cu-dimer site is 1.58 eV, corresponding to the reaction of adsorbed NO_2 with an NH_3 ligand.

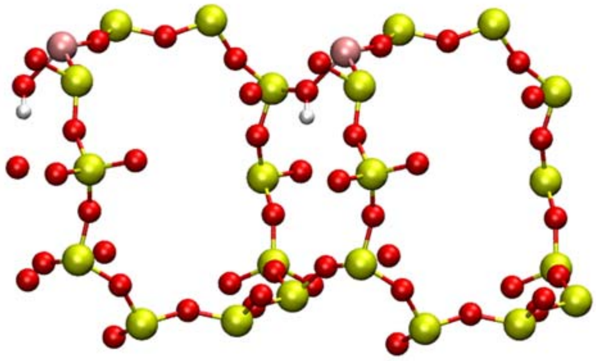

Supplementary Fig. 20 The SSZ-13 zeolite model used in the DFT calculations. Yellow, pink, red and white circles denote Si, Al, O and H atoms, respectively.

Supplementary Table 1 EXAFS fitting parameters for Cu-SSZ-13 exposed to different atmospheres

Sample	Shell	CN	R(Å)	σ^2	ΔE_0	R factor
pretreated	Cu-O	3.7±0.4	1.93±0.01	0.0061	0.5±2.6	0.0124
SSCR reaction	Cu-O(N)	3.5±0.1	1.95±0.01	0.0073	3.7±1.0	0.0124
FSCR reaction	Cu-O(N)	3.7±0.1	1.94±0.01	0.0068	1.9±0.8	0.0124
NO_2-SCR reaction	Cu-O(N)	3.7±0.1	1.95±0.01	0.0068	1.6±0.9	0.0124

Supplementary Table 2 Elemental analysis and physicochemical properties of Cu-SSZ-13 with various Cu contents

Sample	Cu wt.%	Cu/Al	Si/Al	NH_3 storage (mmol/g)	L (mmol/g)	B (mmol/g)	BET (m^2/g)
$Cu_{0.4}$-SSZ-13	0.4	0.03	5.1	2.135	0.328	1.806	626
$Cu_{1.0}$-SSZ-13	1.0	0.08	5.0	2.260	0.662	1.666	575
$Cu_{1.7}$-SSZ-13	1.7	0.14	5.2	2.287	1.145	1.141	566
$Cu_{2.6}$-SSZ-13	2.6	0.22	5.5	2.359	1.361	0.998	592
$Cu_{3.8}$-SSZ-13	3.8	0.31	5.1	2.376	1.523	0.853	544

Supplementary References

1 Xie, L. et al. Inhibitory effect of NO_2 on the selective catalytic reduction of NOx with NH_3 over one-pot-synthesized Cu–SSZ-13 catalyst. *Catal. Sci. Technol.* **4**, 1104-1110 (2014).

2 Shan, Y. et al. Effects of NO_2 Addition on the NH_3-SCR over Small-Pore Cu–SSZ-13 Zeolites with Varying Cu Loadings. *J. Phys. Chem. C* **122**, 25948-25953 (2018).

3 Wang, D. et al. Selective Catalytic Reduction of NOx with NH_3 over a Cu-SSZ-13 Catalyst Prepared by a Solid-State Ion-Exchange Method. *Chem. Commun.* **6**, 1579-1583 (2014).

4 Luo, J. et al. New insights into Cu/SSZ-13 SCR catalyst acidity. Part I: Nature of acidic sites probed by NH_3 titration. *J. Catal.* **348**, 291-299 (2017).

5 Villamaina, R. et al. Speciation of Cu Cations in Cu-CHA Catalysts for NH_3-SCR: Effects of SiO_2/Al_2O_3 Ratio and Cu-Loading Investigated by Transient Response Methods. *ACS Catal.* **9**, 8916-8927 (2019).

6 Grossale, A., Nova, I. & Tronconi, E. Ammonia blocking of the "Fast SCR" reactivity over a commercial Fe-zeolite catalyst for Diesel exhaust aftertreatment. *J. Catal.* **265**, 141-147 (2009).

7 Grossale, A., Nova, I., Tronconi, E., Chatterjee, D. & Weibel, M. NH_3–NO/NO_2 SCR for Diesel Exhausts Aftertreatment: Reactivity, Mechanism and Kinetic Modelling of Commercial Fe- and Cu-Promoted Zeolite Catalysts. *Top. Catal.* **52**, 1837-1841 (2009).

8 Liu, K. et al. Quantitative determination of the Cu species, acid sites and NH_3-SCR mechanism on Cu-SSZ-13 and H-SSZ-13 at low temperatures. Catal. Sci. Technol. **10**, 1135-1150 (2020).

9 Liu, C. et al. *In Situ/Operando* IR and Theoretical Studies on the Mechanism of NH_3–SCR of NO/NO_2 over H–CHA Zeolites. *J. Phys. Chem. C* **125**, 13889-13899 (2021).

10 Grossale, A., Nova, I., Tronconi, E., Chatterjee, D. & Weibel, M. The chemistry of the NO/NO_2–NH_3 "fast" SCR reaction over Fe-ZSM5 investigated by transient reaction analysis. *J. Catal.* **256**, 312-322 (2008).

11 Yeom, Y., Henao, J., Li, M., Sachtler, W. & Weitz, E. The role of NO in the mechanism of reduction with ammonia over a BaNa-Y catalyst. *J. Catal.* **231**, 181-193 (2005).

12 Martini, A. et al. Composition-driven Cu-speciation and reducibility in Cu-CHA zeolite catalysts: a multivariate XAS/FTIR approach to complexity. Chem Sci 8, 6836-6851, doi:10.1039/c7sc02266b (2017).

13 Borfecchia, E. et al. Revisiting the nature of Cu sites in the activated Cu-SSZ-13 catalyst for SCR reaction. Chem. Sci. **6**, 548-563 (2015).

14 Negri, C. et al. Structure and Reactivity of Oxygen-Bridged Diamino Dicopper(II) Complexes in Cu-Ion-Exchanged Chabazite Catalyst for NH_3-Mediated Selective Catalytic Reduction. *J. Am. Chem. Soc.* **142**, 15884-15896 (2020).

15 Pappas, D. K. et al. Methane to Methanol: Structure-Activity Relationships for Cu-CHA. *J. Am. Chem. Soc.* **139**, 14961-14975 (2017).

16 Gao, F., Mei, D., Wang, Y., Szanyi, J. & Peden, C. H. Selective Catalytic Reduction over Cu/SSZ-13: Linking Homo- and Heterogeneous Catalysis. *J. Am. Chem. Soc.* **139**, 4935-4942 (2017).

17 Paolucci, C. et al. Dynamic multinuclear sites formed by mobilized copper ions in NO_x selective catalytic reduction. *Science* **357**, 898-903 (2017).

18 Paolucci, C. et al. Catalysis in a Cage: Condition-Dependent Speciation and Dynamics of Exchanged Cu Cations in SSZ-13 Zeolites. *J. Am. Chem. Soc*. **138**, 6028-6048 (2016).

19 Zhang, Y. et al. Using Transient FTIR Spectroscopy to Probe Active Sites and Reaction Intermediates for Selective Catalytic Reduction of NO on Cu/SSZ-13 Catalysts. *ACS Catal.* **6**, 6137-6145 (2019).

20 Paolucci, C. et al. Isolation of the Copper Redox Steps in the Standard Selective Catalytic Reduction on Cu-SSZ-13. *Angew. Chem. Int. Ed.* **126**, 12022-12027 (2014).

21 Hu, W. et al. On the Redox Mechanism of Low-Temperature NH_3-SCR over Cu-CHA: A Combined Experimental and Theoretical Study of the Reduction Half Cycle. *Angew. Chem. Int. Ed.* **60**, 7197-7204 (2021).

22 Liu, C. et al. In Situ Spectroscopic Studies on the Redox Cycle of NH_3–SCR over Cu-CHA Zeolites. *ChemCatChem*, **12**, 3050-3059 (2020).

23 Bates, S. A. et al. Identification of the active Cu site in standard selective catalytic reduction with ammonia on Cu-SSZ-13. *J. Catal.* **312**, 87-97 (2014).

24 McEwen, J. S. et al. Integrated operando X-ray absorption and DFT characterization of Cu–SSZ-13 exchange sites during the selective catalytic reduction of NOx with NH_3. *Catal. Today* **184**, 129-144 (2012).

25 Sushkevich, V. L., Safonova, O. V., Palagin, D., Newton, M. A. & van Bokhoven, J. A. Structure of copper sites in zeolites examined by Fourier and wavelet transform analysis of EXAFS. *Chem. Sci.* **11**, 5299-5312 (2020).
